# Superior antibody and membrane protein-specific T-cell responses to CoronaVac by intradermal versus intramuscular routes in adolescents

**DOI:** 10.1007/s12519-023-00764-0

**Published:** 2023-12-12

**Authors:** Jaime S. Rosa Duque, Samuel M. S. Cheng, Carolyn A. Cohen, Daniel Leung, Xiwei Wang, Xiaofeng Mu, Yuet Chung, Tsun Ming Lau, Manni Wang, Wenyue Zhang, Yanmei Zhang, Howard H. W. Wong, Leo C. H. Tsang, Sara Chaothai, Tsz Chun Kwan, John K. C. Li, Karl C. K. Chan, Leo L. H. Luk, Jenson C. H. Ho, Wing Yan Li, Amos M. T. Lee, Jennifer H. Y. Lam, Sau Man Chan, Wilfred H. S. Wong, Issan Y. S. Tam, Masashi Mori, Sophie A. Valkenburg, Malik Peiris, Wenwei Tu, Yu Lung Lau

**Affiliations:** 1 Department of Paediatrics and Adolescent Medicine, The University of Hong Kong, Hong Kong, China; 2 School of Public Health, The University of Hong Kong, Hong Kong, China; 3 HKU-Pasteur Research Pole, School of Public Health, The University of Hong Kong, Hong Kong, China; 4 Research Institute for Bioresources and Biotechnology, Ishikawa Prefectural University, Nonoichi, Japan; 5 Department of Microbiology and Immunology, Peter Doherty Institute for Infection, and Immunity, University of Melbourne, Melbourne, VIC Australia; 6 Center for Immunology and Infection C2i, Hong Kong, China

**Keywords:** CoronaVac, Coronavirus disease 2019, Immunity, Intradermal, Vaccine

## Abstract

**Background:**

Optimising the immunogenicity of COVID-19 vaccines to improve their protection against disease is necessary. Fractional dosing by intradermal (ID) administration has been shown to be equally immunogenic as intramuscular (IM) administration for several vaccines, but the immunogenicity of ID inactivated whole severe acute respiratory syndrome coronavirus 2 (SARS-CoV-2) at the full dose is unknown. This study (NCT04800133) investigated the superiority of antibody and T-cell responses of full-dose CoronaVac by ID over IM administration in adolescents.

**Methods:**

Participants aged 11–17 years received two doses of IM or ID vaccine, followed by the 3rd dose 13–42 days later. Humoral and cellular immunogenicity outcomes were measured post-dose 2 (IM-CC versus ID-CC) and post-dose 3 (IM-CCC versus ID-CCC). Doses 2 and 3 were administered to 173 and 104 adolescents, respectively.

**Results:**

Spike protein (S) immunoglobulin G (IgG), S-receptor-binding domain (RBD) IgG, S IgG Fcγ receptor IIIa (FcγRIIIa)-binding, SNM [sum of individual (S), nucleocapsid protein (N), and membrane protein (M) peptide pool]-specific interleukin-2 (IL-2)^+^CD4^+^, SNM-specific IL-2^+^CD8^+^, S-specific IL-2^+^CD8^+^, N-specific IL-2^+^CD4^+^, N-specific IL-2^+^CD8^+^ and M-specific IL-2^+^CD4^+^ responses fulfilled the superior and non-inferior criteria for ID-CC compared to IM-CC, whereas IgG avidity was inferior. For ID-CCC, S-RBD IgG, surrogate virus neutralisation test, 90% plaque reduction neutralisation titre (PRNT90), PRNT50, S IgG avidity, S IgG FcγRIIIa-binding, M-specific IL-2^+^CD4^+^, interferon-γ^+^CD8^+^ and IL-2^+^CD8^+^ responses were superior and non-inferior to IM-CCC. The estimated vaccine efficacies were 49%, 52%, 66% and 79% for IM-CC, ID-CC, IM-CCC and ID-CCC, respectively. The ID groups reported more local, mild adverse reactions.

**Conclusion:**

This is the first study to demonstrate superior antibody and M-specific T-cell responses by ID inactivated SARS-CoV-2 vaccination and serves as the basis for future research to improve the immunogenicity of inactivated vaccines.

**Graphical abstract:**

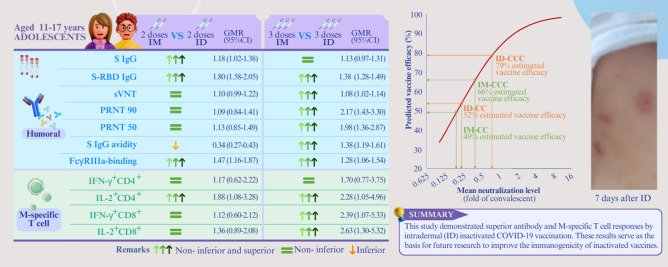

**Supplementary Information:**

The online version contains supplementary material available at 10.1007/s12519-023-00764-0.

## Introduction

The coronavirus disease 2019 (COVID-19) pandemic caused by severe acute respiratory syndrome coronavirus 2 (SARS-CoV-2) remains a major global public health concern. Although hospitalizations were rarer for adolescents, severe disease still occurred [1]. During an outbreak by Omicron variants in Hong Kong, China, in 2022, pediatric hospitalizations increased, with acute neurological and respiratory complications, multisystem inflammatory syndrome in children, long COVID and mental health issues being reported in children and young people, outcomes that can be ameliorated by vaccination [1–5].

Initial landmark trials demonstrated that the nucleoside-modified mRNA vaccine BNT162b2 and inactivated whole-virus vaccine CoronaVac had about 90%–95% and 50%–85% efficacies against symptomatic COVID-19 in persons aged ≥ 16 and ≥ 18 years old, respectively [6–8]. The efficacy of BNT162b2 in another phase 3 study for 12- to 15-year-old adolescents was 100% [9]. These vaccines, with efficacies > 50%, have been approved for emergency use since 2021. In a phase 2 trial on CoronaVac for adolescents, two doses induced 100% seroconversion in those 12–17 years old [10]. However, the real-life effectiveness of CoronaVac in the prevention of hospitalisation for adolescents was lower, at about 90% in Chile and Hong Kong of China, and it is further reduced against infection [5, 11, 12].

As inactivated vaccines, namely, CoronaVac, have been amongst the most widely used COVID-19 vaccines for individuals ≥ 3 years old globally, our group performed a humoral and cellular immuno-bridging study during the early phase of vaccine availability and found non-inferior immunogenicity for CoronaVac in adolescents compared to adults [13, 14]. However, CoronaVac induced lower antibody responses than BNT162b2 in adolescents [14]. These findings were consistent with the observation that the efficacy of CoronaVac against infection appeared lower than that of BNT162b2 in separate pivotal clinical trials [6–8].

Moreover, emerging variants of concern (VOCs), such as various Omicron subvariants, developed mutations at numerous sites that allow neutralising antibody escape for previously infected or vaccinated individuals, further raising concerns for reduced efficacies. In our recent study, the immunogenicity of CoronaVac against Omicron was markedly lower than that of the wild-type (WT) strain in adolescents [15]. As a result of immune evasion by these VOCs and waning antibodies, inclusion of the third dose as part of the primary series of CoronaVac had been recommended in Hong Kong of China and Singapore for most age groups. It is apparent that all feasible strategies for optimising immunological responses to CoronaVac need to be urgently explored.

Intradermal (ID) vaccination has been shown to be safe and can enhance immunogenicity compared to the intramuscular (IM) route [16]. Introduction of viral antigens and adjuvant into the skin activates resident innate cells, including dermal CD14^+^ dendritic cells, Langerhans cells and mast cells that secrete cytokines, cross present to CD8^+^ T cells and prime CD4^+^ T cells to induce switching of naïve B cells into immunoglobulin G (IgG)- and IgA-producing isotypes, with the major immune correlates that consist of IgG and cellular responses deriving from peripheral vaccination or natural infection, whereas higher IgA production is associated with infection or vaccines that act at mucosal sites [17–23]. Fractional vaccine dosing has been studied extensively for ID to mitigate vaccine inequity during supply shortages or unaffordable costs, especially for the inactivated poliovirus, hepatitis B and human papillomavirus vaccines, which showed similar humoral immunogenicity as full doses of IM [24–26].

Inactivated influenza vaccines, produced by similar technology as CoronaVac, also induced similar IgG titers with fractional dosing by the ID as the full IM dose [16]. In two separate studies, our group showed that children 6 months to 17 years old who received inactivated influenza vaccination intradermally at one-fifth of the IM dose developed similar antibody responses to the conventional IM dose [27, 28]. For full ID dosing, two trials found superior geometric mean (GM) hemagglutination inhibition antibody titers and sero-protection rates compared to IM of the inactivated influenza vaccines in older adults [29, 30].

Several investigators recently compared fractional ID ChAdOx1/AZD-1222 with BNT162b2 boosters after two IM injections of CoronaVac [31, 32]. Both ID boosters raised IgG levels against SARS-CoV-2 [31, 32]. Fractional ID ChAdOx1/AZD-1222 induced similar antibody and T-cell responses as the full IM booster [31]. However, ID use of full doses of COVID-19 vaccines has not been studied thus far. Since mRNA vaccination appears to induce higher antibody responses and efficacies than the inactivated vaccine, optimization of humoral immunity using the ID method would be worthwhile to explore, especially against a pathogen capable of causing millions of deaths [1, 5–8, 14]. Based on the collective available scientific data, we postulated that ID with the full dose of CoronaVac can induce greater immunogenicity against SARS-CoV-2 than the currently recommended IM. This study aimed to compare the reactogenicity of 2 and 3 full doses of CoronaVac between ID and IM and show superior immunogenicity with ID for adolescents 11–17 years old. The current study presents a pre-specified interim analysis of the immunogenicity against WT and Omicron SARS-CoV-2, reactogenicity and safety results at 1 month after 2 and 3 doses of CoronaVac.

## Methods

### Study design

This registered study is part of the COVID-19 Vaccination in Adolescents and Children (Department of Health, Hong Kong, China; clinical trial certificate 101,894; clinicaltrials.gov NCT04800133) that investigates immuno-bridging for BNT162b2 and CoronaVac in adolescents and children, as previously described [14, 15, 33]. The current pre-specified interim analysis aims to demonstrate the superiority in immunogenicity of the ID compared to the IM route of administration for CoronaVac in adolescents 11–17 years old and reports on the reactogenicity between ID and IM. The University of Hong Kong/Hong Kong West Cluster Hospital Authority Institutional Review Board (UW21-157) approved the research procedures, which were in compliance with the October 2013 Declaration of Helsinki principles.

### Participants

Recruitment targeted 11- to 17-year-old adolescents residing in Hong Kong, China, who were healthy or in stable condition. Potential participants were recruited using advertisements posted in schools and mass media. The exclusion criteria for this analysis included a known history of COVID-19 [by self-reporting at any of the four study visits or baseline spike protein (S)-receptor-binding domain (RBD) IgG or open reading frame 8 (ORF8) IgG positivity at any visit], severe allergy, neuropsychiatric conditions, immunocompromised states, transfusion of blood products within 60 days, haemophilia, pregnancy or breastfeeding (see Supplementary protocol and statistical analysis plan for details).

### Procedures

Study doctors obtained informed assent from eligible participants and consent from their respective parents or legally acceptable representatives. The skin superficial to the deltoid muscle was cleansed with 70% weight/volume isopropyl alcohol before using standard 1 mL (KDL Medical, Shanghai, China) or MicronJet600 (NanoPass Technologies, Ness Ziona, Israel) needles for IM or ID, respectively, at a Hong Kong Community Vaccination Center (CVC) (Supplementary video) [34]. The dosage of CoronaVac was 0.5 mL (equivalent to 600 SU, or 3 µg, of the whole-virus antigen of the inactivated SARS-CoV-2 CZ02 strain) for each injection, with a total of 3 separate doses given IM or ID. Doses 2 and 3 were given 28–35 days and 84 days after dose 1, respectively. Whole blood was obtained before doses 1 (baseline), 2 (C), 3 (CC) and post dose 3 (CCC) (see “Analysis populations” in “Statistical analyses”).

### Safety and reactogenicity data collection

Participants remained at the CVC for observation by the study nurse and doctor for at least 15 minutes after each vaccine injection. Participants were required to report pre-specified adverse reactions (ARs) in an online or handwritten diary for the following seven days, as previously described [14, 15]. They were encouraged to capture photos of their sites of injection for at least seven days and until resolution of local reactions, followed by uploading onto our online diary website. Unsolicited adverse events (AEs), such as hospitalisation, life-threatening illnesses, disabilities, deaths, birth defects of offspring and breakthrough COVID-19, are monitored for three years. These AEs were reviewed by study physicians, who assessed the probability of a causal relationship with the study vaccination.

### S-RBD IgG, surrogate virus neutralisation assay and plaque reduction neutralisation titre

Peripheral blood was collected into clot activator vacutainer tubes, separated into serum, and stored at  – 80 °C. Sera were heat-inactivated (HI) at 56 °C for 30 minutes prior to testing. The SARS-CoV-2 S-RBD IgG enzyme-linked immunosorbent assay (ELISA) and plaque reduction neutralisation titre (PRNT) have been validated in our previous publications [14, 15, 33, 35]. The surrogate virus neutralisation assay (sVNT) was carried out according to the manufacturer’s instructions (GenScript Inc., Piscataway, USA) and our previous experiments, which have been validated [14, 15, 33, 35].

Briefly, S-RBD IgG ELISA plates were coated overnight with 100 ng/well of purified recombinant S-RBD in phosphate buffered saline (PBS), and then 100 μL of Chonblock blocking/sample dilution (CBSD) ELISA buffer (Chondrex Inc., Redmond, USA) was added. The incubation period of this mixture at room temperature (RT) was 2 hours. Serum was tested at a dilution of 1:100 in CBSD ELISA buffer and then added to the wells for 2 hours at 37 °C. After washing with PBS that contained 0.2% Tween 20, horseradish peroxidase (HRP)-conjugated goat anti-human IgG (1:5000, Thermo Fisher Scientific) was added for 1 hour at 37 °C and then washed five times with PBS containing 0.2% Tween 20. HRP substrate (Ncm TMB One, New Cell & Molecular Biotech Co. Ltd, China) at 100 μL was added for 15 minutes. This reaction was stopped by 50 μL of 2 mol/L H_2_SO_4_. The optical density (OD) was analysed in a Sunrise absorbance microplate reader (Tecan, Männedorf, Switzerland) at 450 nm wavelength. Each OD reading subtracted the background OD in PBS-coated control wells with the participant’s serum. Values at or above an OD450 of 0.5 were considered positive, whilst values below were imputed as 0.25 [14, 15, 33].

The sVNT was performed using 10 μL of each serum and positive and negative controls, which were diluted at 1:10 and mixed with an equal volume of HRP conjugated to the WT SARS-CoV-2 S-RBD (6 ng), and these were incubated for 30 minutes at 37 °C. Then, 100 μL of each sample was added to microtiter plate wells coated with angiotensin-converting enzyme-2 receptor [14, 15, 33]. This plate was sealed for 15 minutes at 37 °C, washed with wash-solution, tapped dry, and then 100 μL of 3,3′,5,5′-tetramethylbenzidine was added. This mixture was incubated in the dark at RT for 15 minutes. The reaction was terminated with 50 μL of stop solution, and the absorbance was read at 450 nm in a microplate reader. After confirmation that the positive and negative controls provided the recommended OD450 values, the %inhibition of each serum was calculated as (1-sample OD value/negative control OD value) × 100%. Inhibition (%) of at least 30%, the limit of quantification, was regarded as positive, whilst values below 30% were imputed as 10% [14, 15, 33].

The PRNT was performed in duplicate in a biosafety level 3 facility [14, 35]. Serial dilutions of serum from 1:10 to at least 1:320 were incubated with approximately 30 plaque-forming units of the WT SARS-CoV-2 BetaCoV/Hong Kong/VM20001061/2020 virus in culture plates (Techno Plastic Products AG, Trasadingen, Switzerland) for 1 hour at 37 °C [14, 15, 33]. These virus–serum mixtures were added to Vero E6 cell monolayers and incubated for 1 hour at 37 °C in a 5% CO_2_ incubator. The plates were overlaid with 1% agarose in cell culture medium and incubated for three days, and then the plates were fixed and stained. Antibody titers were defined as the reciprocal of the highest serum dilution that resulted in the more stringent cutoff of > 90% (PRNT90) or > 50% (PRNT50) reduction in the number of plaques. Values below the lowest dilution tested, which was 10, were imputed as 5, whilst those above 320 were imputed as 640 [14, 15, 33].

### Spike protein immunoglobulin G avidity and Fcγ receptor IIIa-binding

S IgG avidity and Fcγ receptor IIIa (FcγRIIIa)-binding assays were performed as previously described, with the addition of Omicron BA.2 [14, 15, 33, 36]. In brief, plates (Nunc MaxiSorp, Thermo Fisher Scientific) were coated with 250 ng/mL WT (AcroBiosystems) or Omicron BA.2 (AcroBiosystems) SARS-CoV-2 S protein for IgG and IgG avidity assessments, 500 ng/mL WT (Sinobiological) or Omicron BA.2 (AcroBiosystems) S for FcγRIIIa-binding detection, or 300 ng/mL ORF8 (Masashi Mori, Ishiwaka University, Japan) at 37 °C for 2 hours [37]. The protein for S IgG was diluted in PBS. The plates were blocked with 1% foetal bovine serum (FBS) in PBS for 1 hour and incubated with 1:100 HI serum diluted in 0.05% Tween 20/0.1% FBS in PBS for 2 hours at RT prior to rinsing. For antibody avidity, plates were washed thrice with 8 mol/L urea before incubation for 2 hours with IgG-HRP (1:5000; G8-185, BD). HRP was revealed by stabilised hydrogen peroxide and tetra-methyl-benzidine (R & D systems) for 20 minutes and stopped with 2 mol/L H_2_SO_4_ before analysis with an absorbance microplate reader at 450 nm wavelength (Tecan Life Sciences). For those with a positive S IgG value, the IgG avidity index was calculated by the ratio of the OD450 values after to before washing of the plates, censored at 100%. FcγRIIIa-binding antibodies were detected after incubation with HI serum at a 1:50 dilution for 1 hour at 37 °C and then with biotinylated FcγRIIIa-V158, which was expressed in-house (from Mark Hogarth and Bruce Wines, Burnet Institute, Australia), at 100 ng/mL for 1 hour at 37 °C. Streptavidin-HRP (1:10,000, Pierce) was added for detection of S-specific FcγRIIIa-V158-binding antibodies. OD450 values at or above the respective limits of detection (LODs) were considered positive, whilst values below were imputed as 0.5 of the LOD [14, 15, 33].

### T-cell responses

Density gradient separation was performed to isolate peripheral blood mononuclear cells (PBMCs) from whole blood, which was frozen in liquid nitrogen [14, 15, 33]. Subsequently, thawed PBMCs were rested for 2 hours in RPMI medium supplemented with 10% human AB serum. The cells were stimulated with sterile double-distilled water (ddH_2_O) or 1 µg/mL overlapping peptide pools representing the WT SARS-CoV-2 S, N (nucleocapsid) and M (membrane) proteins or Omicron B.1.1.529/BA.1 S mutation pool and WT reference pool, BA.1 N mutation pool and WT N reference pool, BA.1 M mutation pool and WT M reference pool (Miltenyi Biotec, Bergisch Gladbach, Germany) (synthesised by ChinaPeptides Co., Ltd, as previously described) for 16 hours in 1 µg/mL anti-CD28 and anti-CD49d costimulatory antibodies (clones CD28.2 and 9F10, Biolegend, San Diego, USA) [14, 15, 33]. This mixture was stimulated for 2 hours, followed by the addition of 10 µg/mL brefeldin A (Sigma, Kawasaki, Japan) [14, 15, 33, 38]. The cells were then washed and subjected to immunostaining with a fixable viability dye (eBioscience, Santa Clara, USA, 1:60) and antibodies against CD3^+^ (HIT3a, 1:60), CD4^+^ (OKT4, 1:60), CD8^+^ (HIT8a, 1:60), interferon-γ (IFN-γ) (B27, 1:15) and interleukin-2 (IL-2) (MQ1-17H12, 1:15) (Biolegend, San Diego, USA). Flow cytometry (LSR II with FACSDiva version 8.0, BD Biosciences, Franklin Lakes, USA), analysed by FlowJo version 10 software (BD, Ashland, USA), was used for data acquisition. The gating strategy followed our past publication as described [14]. Antigen-specific T-cell results were finalised after subtracting the background (ddH_2_O) data and presented as the percentage of CD4^+^ or CD8^+^ T cells [14, 15, 33, 39]. The T-cell response against a single peptide pool was considered positive when the frequency of cytokine-expressing cells was higher than 0.005% and the stimulation index was higher than 2, whilst negative values were imputed as 0.0025% [14, 15, 33]. Total T-cell responses against S, N and M peptide pools were added together, which used a cutoff of 0.01% [14, 15, 33].

### Outcomes

The primary outcomes in this interim analysis were humoral immunogenicity (S IgG and S-RBD IgG levels, sVNT %inhibition, 90% and 50% PRNT titers, S IgG avidity and FcγRIIIa-binding) and cellular immunogenicity markers (S-, N- and M-specific IFN-γ^+^ and IL-2^+^ CD4^+^ and CD8^+^ T-cell responses measured by the flow-cytometry-based intracellular cytokine staining assay) 13–42 days after doses 2 and 3 of CoronaVac. The primary reactogenicity outcomes included pre-specified ARs and reported antipyretic use during the seven days following each vaccine injection.

Omicron humoral and cellular immunogenicity results were secondary outcomes. Regarding safety, the secondary outcomes were unsolicited AEs within 28 days after each vaccine injection and serious AEs (SAEs) during the entire study period. The supplementary protocol and the statistical analysis plan described other secondary outcomes that were not pertinent to this interim analysis, such as specific assessments for participants with known chronic illnesses.

### Statistical analyses

#### Power analyses and sample size estimation

For the primary immunogenicity objectives, when comparing the peak GM immunogenicity outcomes of seroconversion rate or AEs for CoronaVac ID with that of IM administration in adolescents aged 11–17 years, a sample size of 50 in each group would assure that a two-sided test with α = 0.05 has 97% power to detect an effect size with a Cohen’s *d* value = 0.78 or a difference of 0.51 after natural logarithmic transformation between the two groups, with a standard deviation of 0.65 within each group. We aimed to recruit 60 participants for each group of IM and ID administrations to accommodate for potential attrition or protocol deviation. However, the performance of assays requiring large blood volumes was omitted for a few younger, small-sized adolescents, who could provide limited amounts of blood. In terms of the proportion of participants with a positive result in immunogenicity outcomes or ARs, 50 adolescents would yield a 95% chance to detect the true value within ± 11 percentage points of the measured percentage, assuming a prevalence of 80%. G*Power (Heinrich-Heine-Universität Düsseldorf, Düsseldorf, Germany) and Sampsize (sampsize.sourceforge.net) were used for these power analyses.

#### Analysis populations

The primary analysis of humoral and cellular immunogenicity outcomes was performed in healthy adolescent participants who received IM or ID injections of CoronaVac on a per-protocol basis. The evaluable analysis population included participants who were generally healthy and remained uninfected during study visits (based on self-reporting, ORF8 IgG negativity and negative baseline S-RBD IgG), had no major protocol deviations, received dose 3 at least 84 days after dose 1, had blood sampling within the evaluable window for post-dose 1 (no more than 3 days earlier or later than day 28 and before dose 2), post-dose 2 (within day 13–42 post-dose 2 and before any further doses), and within days 13–42 post-dose 3 and had valid results for the relevant analysis and time points (see Supplementary protocol). The expanded analysis population included similar criteria as the evaluable analysis population except for the requirement of a valid immunogenicity result for the particular analysis at least 14 days post-dose 1 but before dose 2 and between 7 and 56 days post-dose 2 (see Supplementary protocol). The superiority and hypothesis testing for primary immunogenicity outcomes included participants aged 11–17 years in the adolescent groups who received IM or ID injections of CoronaVac at doses 1–3.

#### Statistical tests

Immunogenicity outcome data below the cutoff were imputed with half the cutoff value. GMs were calculated for each immunogenicity outcome, time point and group. GM ratios (GMRs) were calculated as exponentiated differences between the means of the natural logarithmic-transformed immunogenicity outcomes between groups. The GMRs were reported with a two-sided 95% confidence interval (CI) for testing the superiority hypothesis with the lower bound of the 95% CI for GMR > 1. Additionally, confirmation of the superiority results was performed in the expanded analysis population. Simultaneously, we conducted a non-inferiority analysis at the non-inferiority margin of 0.60 for immunogenicity outcomes since it was possible that superiority for a few immunogenicity outcomes would not be satisfied. By convention, the results were regarded as inconclusive if both non-inferiority and inferiority were not met. Comparisons of immunogenicity outcomes between groups were performed with unpaired *t* tests after natural logarithmic transformation. The proportion of participants with a positive result was reported in percentages, with a two-sided 95% CI derived from the Clopper–Pearson method. Fisher’s exact test was used for comparisons of proportions between groups.

Reactogenicity and safety were assessed in the participants who remained generally healthy, uninfected and contributed any AR or AE data after the 3 doses and before the study database was locked for the current interim analysis in these adolescent groups who received IM or ID CoronaVac that comprised the healthy safety population. For the primary reactogenicity analysis, the proportions of participants reporting each AR at the maximum severity and antipyretic use within seven days after each vaccine injection were reported in percentages, with the 95% CI derived using the Clopper–Pearson method. ARs of all severity and antipyretic use were compared between vaccine routes of administration by Fisher’s exact test. The incidences of AEs by severity and SAEs that were reported by the post-dose 3 study visit (28 days post-dose 3) were presented as counts and events per participant by the vaccine route of administration. Data analyses and graphing were performed using GraphPad Prism (version 9.4.0). Two-sided 95% CIs are presented for all outcomes unless otherwise stated.

#### Vaccine efficacy estimates

Vaccine efficacies were estimated as a secondary objective by correlations with neutralising antibody titers, as described in our previous publication [14, 15, 40]. In brief, GMTs of PRNT50 in the evaluable analysis populations were divided from that of 102 convalescent sera collected on days 28–59 post-onset of illness in patients aged ≥ 18 years to calculate the mean neutralising levels. The best fit of the logistic model, generated from the online plot digitizer tool (https://automeris.io/WebPlotDigitizer/, version 4.5), was used to extrapolate a single point vaccine effectiveness estimate for each route of vaccine administration.

## Results

### Study participants

A total of 185 adolescents aged 11–17 years received at least 1 dose of IM or ID CoronaVac at the screening visit (V1) from 27 April 2021 to 06 August 2022 (Supplementary Fig. 1). There were 185 and 178 participants who returned for subsequent follow-up visits 2 (V2) and 3 (V3), respectively, and those who attended V3 were included in the reactogenicity and safety analyses (healthy safety population; see Supplementary protocol and statistical analysis plan; Supplementary Fig. 1). The evaluable analysis population included those uninfected based on the clinical history obtained and negative ORF8 IgG (a serological marker of past natural SARS-CoV-2 infection) at every visit, who had negative baseline S-RBD IgG, no major protocol deviations and a valid immunogenicity result (Supplementary Fig. 1). Of the 173 who completed the 2-dose series (IM-CC and ID-CC), 104 received 3 vaccine doses (IM-CCC and ID-CCC) and returned for the follow-up visit (V4), all within the evaluable intervals (Supplementary Fig. 1). A total of 119 IM-CC and 54 ID-CC participants were included in the evaluable analysis population, with 60 IM and 44 ID recipients for 3 doses. We confirmed these findings by performing an analysis with the expanded analysis population that consisted of 119 IM-CC, 59 ID-CC, 82 IM-CCC and 43 ID-CCC participants who had wider time intervals of vaccination and blood sampling (Supplementary Fig. 1). There was an even distribution of demographic characteristics between the IM and ID groups (Supplementary Table 1).

### Humoral immunogenicity analyses between IM and ID administrations

**Fig. 1:**
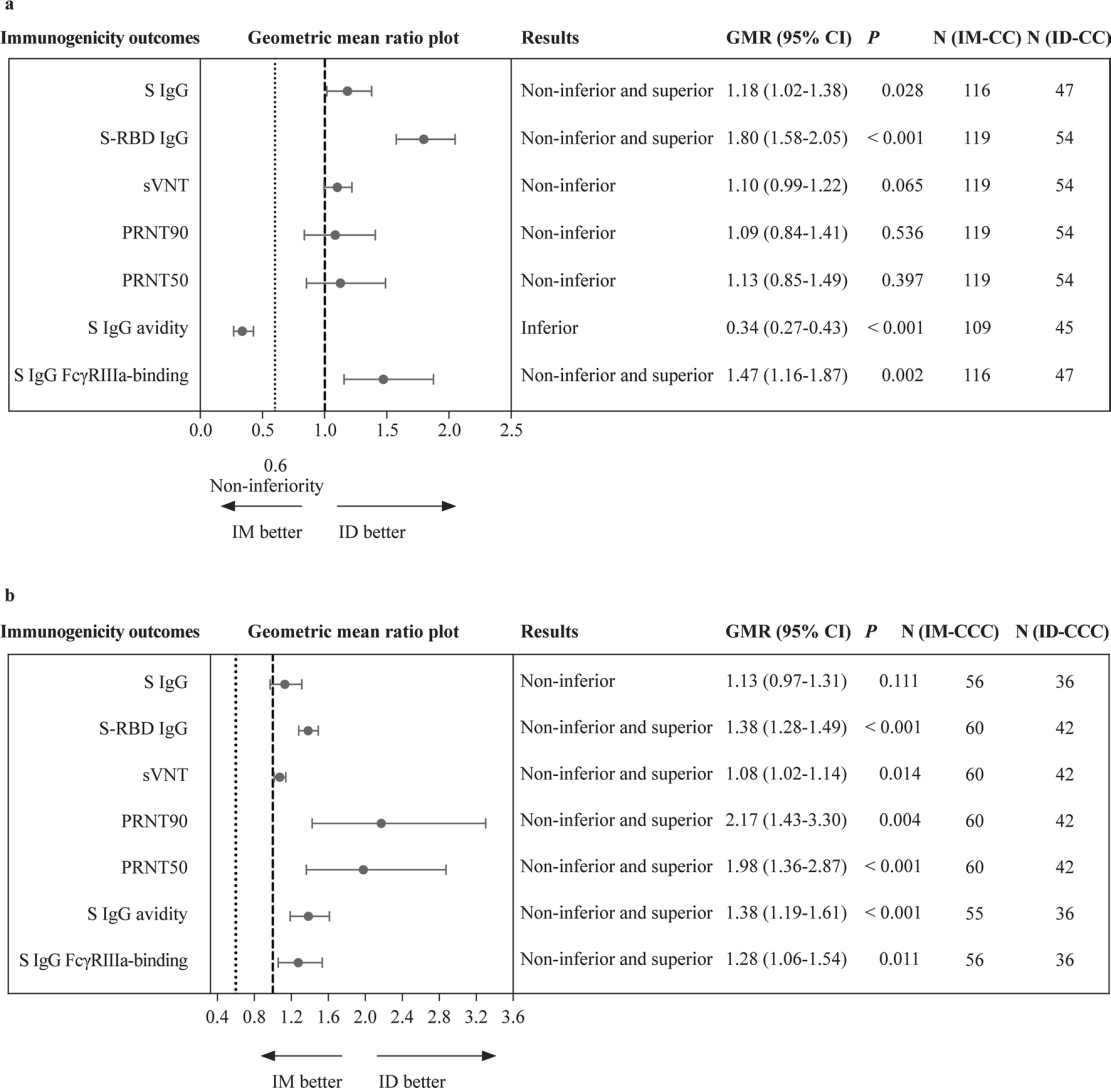
Superiority and non-inferiority hypothesis testing of humoral immunogenicity against wild type SARS-CoV-2 post-dose 2 and post-dose 3 of vaccination in the evaluable analysis population. Adolescents receiving 2 doses of CoronaVac administered intramuscularly (IM-CC) or intradermally (ID-CC) (**a**) and 3 doses of CoronaVac administered intramuscularly (IM-CCC) or intradermally (ID-CCC) (**b**) were tested for humoral immunogenicity outcomes. Dots and error bars show GMR estimates and two-sided 95% CI, respectively. *SARS-CoV-2* severe acute respiratory syndrome coronavirus 2, *GMR* geometric mean ratio, *ID* intradermal, *IM* intramuscular, *N* nucleocapsid protein, *S* spike protein, *RBD* receptor-binding domain, *sVNT* surrogate virus neutralisation test, *PRNT* plaque reduction neutralisation titre, *FcγRIIIa* Fcγ receptor IIIa, *IgG* immunoglobulin G, *CI* confidence interval

The primary humoral immunogenicity outcomes in this study were SARS-CoV-2 S IgG, S-RBD IgG by ELISA, sVNT, PRNT, S IgG avidity and S IgG FcγRIIIa-binding on ELISA performed for healthy, uninfected adolescents 13–42 days after dose 2 or 3 of CoronaVac by IM or ID. Evaluable IM-CC achieved 96.6% S-RBD IgG seropositivity, with a GM OD450 of 1.20 and sVNT inhibition of 71.2% post-dose 2 (Table 1). A total of 100.0% of evaluable ID-CC participants had positive S-RBD IgG, with a GM OD450 value of 2.16 and sVNT inhibition of 78.4%. The GM for PRNT90 was 9.83 and 10.7 after IM-CC and ID-CC, respectively. The GM for PRNT50 against WT was 26.8 and 30.2 after IM-CC and ID-CC, respectively. After IM-CC and ID-CC, the GM avidity was 20.5% and 6.95%, and the GM OD450 of S IgG FcγRIIIa-binding was 0.749 and 1.10, respectively. Compared to IM-CC, humoral responses, when measured by S IgG, S-RBD IgG and S IgG FcγRIIIa-binding, satisfied the superior and non-inferior criteria for evaluable ID-CC (Fig. 1a). ID-CC mounted non-inferior humoral responses by sVNT, PRNT90, PRNT50 and inferior S IgG avidity.

**Table 1:** Humoral immunogenicity outcomes against wild type SARS-CoV-2 post-dose 2 and post-dose 3 of CoronaVac in the evaluable analysis population

Variables	Intramuscular		Intradermal	
	2 doses	3 doses	2 doses	3 doses
S IgG on ELISA				
* n*	116	56	47	36
GM OD450 value (95% CI)	0.536 (0.493–0.582)	0.930 (0.830–1.040)	0.634 (0.562–0.716)	1.050 (0.983–1.120)
%positive (≥ LOD at 0.3)	94.0	98.2	95.7, *P* > 0.9999	100.0, *P* > 0.9999
S-RBD IgG on ELISA				
* n*	119	60	54	42
GM OD450 value (95% CI)	1.200 (1.100–1.310)	1.770 (1.680–1.870)	2.160 (2.040–2.290)	2.450 (2.330–2.570)
%positive (≥ LOD at 0.5)	96.6	100.0	100.0, *P* = 0.311	100.0, *P* > 0.9999
S-RBD ACE2-blocking antibody on sVNT				
* n*	119	60	54	42
GM% inhibition (95% CI)	71.2 (66.7–76.0)	84.9 (81.3–88.6)	78.4 (74.6–82.5)	91.3 (88.3–94.4)
%positive (≥ LOQ at 30%)	96.6	100.0	100.0, *P* = 0.311	100.0, *P* > 0.9999
Neutralising antibody on PRNT				
* n*	119	60	54	42
GM PRNT90 (95% CI)	9.83 (8.67–11.10)	17.80 (13.70–23.30)	10.70 (8.09–14.10)	38.70 (27.60–54.20)
%positive (≥ LOD at 10)	65.6	78.3	61.1, *P* = 0.610	97.6,* P* = 0.007
GM PRNT50 (95% CI)	26.80 (23.00–31.10)	55.30 (43.20–70.70)	30.20 (23.50–38.80)	110.00 (82.50–145.00)
%positive (≥ LOD at 10)	96.6	100.0	98.2, *P* > 0.9999	100.0, *P* > 0.9999
S IgG avidity on ELISA				
* n*	109	55	45	36
GM avidity index (95% CI)	20.50 (19.10–22.10)	38.50 (34.60–42.80)	6.95 (5.03–9.60)	53.30 (48.30–58.80)
S IgG FcγRIIIa-binding on ELISA				
* n*	116	56	47	36
GM OD450 value (95% CI)	0.749 (0.649–0.864)	1.410 (1.210–1.630)	1.100 (0.955–1.270)	1.790 (1.710–1.880)
%positive (≥ LOD at 0.28)	87.1	98.2	95.7, *P* = 0.156	100.0, *P* > 0.9999

*SARS-CoV-2* severe acute respiratory syndrome coronavirus 2, *S* spike protein, *N* nucleocapsid protein, *IgG* immunoglobulin G, *ELISA* enzyme-linked immunosorbent assay, *GM* geometric mean, *OD* optical density, *CI* confidence interval, *LOD* limit of detection, *LOQ* limit of quantification, *RBD* receptor-binding domain, *ACE-2* angiotensin-converting enzyme-2, *sVNT* surrogate virus neutralisation test, *PRNT* plaque reduction neutralisation titre, *PRNT90* 90% plaque reduction neutralisation titre, *PRNT50* 50% plaque reduction neutralisation titre, *Fc*γ*RIIIa* Fc gamma receptor III-a. *P* values compare the proportion of positive responses between intramuscular and intradermal administration by Fisher’s exact test

Since IM-CCC had been recommended as the primary vaccination series in Hong Kong, China and Singapore, this regimen was also compared with ID-CCC. Evaluable IM-CCC achieved 100.0% S-RBD IgG seropositivity, with GM OD450 and sVNT inhibition of 1.77 and 84.9% post-dose 3, respectively (Table 1). A total of 100.0% of evaluable ID-CCC had positive S-RBD IgG, with a GM OD450 value of 2.45 and sVNT inhibition of 91.3%. Neutralisation titers demonstrated GMs of 17.8 and 38.7 for PRNT90 after IM-CCC and ID-CCC, respectively. The GM for PRNT50 was 55.3 and 110 after IM-CCC and ID-CCC, respectively. GM avidity was 38.5% and 53.3%, and the GM OD450 results of S IgG FcγRIIIa-binding were 1.41 and 1.79 after IM-CCC and ID-CCC, respectively. ID-CCC satisfied the superior and non-inferior criteria by S-RBD IgG, sVNT, PRNT90, PRNT50, S IgG avidity and S IgG FcγRIIIa-binding but not by S IgG, which satisfied the non-inferiority criterion only (Fig. 1b). For both groups, baseline (pre-vaccination) values were similar, all of which were below the levels of detection and rose after each vaccine dose (Supplementary Table 2; Supplementary Fig. 2). Superiority and non-inferiority testing in the expanded analysis populations for post-doses 2 and 3 were analogous to these results (Supplementary Table 3; Supplementary Fig. 3).

### Cellular immunogenicity analyses between IM and ID administrations

The primary cellular immunogenicity outcomes for this study were IFN-γ^+^ and IL-2^+^CD4^+^ and CD8^+^ T-cell responses to S, N and M after IM-CC and ID-CC, which were analysed using intracellular cytokine staining by flow cytometry. For the 60 CC-IM and 48 CC-ID evaluable adolescents, more than half of the participants had detectable responses for S-specific IFN-γ^+^CD4^+^ or IL-2^+^CD4^+^ T cells after 2 ID or IM doses (Table 2). There were 45.8%–52.1% of those with IFN-γ^+^CD8^+^ and IL-2^+^CD8^+^ T-cell responses to S after 2 doses of either administration route. The remainder of the T-cell responses to the peptide pools S, N, M and SNM (sum of individual S, N, and M peptide pools) after IM-CC, ID-CC, IM-CCC and ID-CCC are also shown in Table 2.

**Table 2:** Cellular immunogenicity outcomes against wild type SARS-CoV-2 S, N and M peptide pools post-dose 2 and post-dose 3 of CoronaVac in the evaluable analysis population

Variables	Intramuscular		Intradermal	
	2 doses	3 doses	2 doses	3 doses
Total SNM-specific T-cell responses on flow cytometry				
* n*	60	58	48	40
GM% IFN-γ^+^CD4^+^ T cells (95% CI)	0.058 (0.040–0.083)	0.066 (0.041–0.106)	0.107 (0.063–0.183)	0.102 (0.053–0.197)
%positive (≥ cut-off at 0.0075%)	83.3	74.1	81.3, *P* = 0.804	77.5,* P* = 0.813
GM% IL-2^+^CD4^+^ T cells (95% CI)	0.040 (0.030–0.052)	0.073 (0.049–0.109)	0.112 (0.068–0.183)	0.143 (0.077–0.264)
%positive (≥ cut-off at 0.0075%)	83.3	79.3	79.2, *P* = 0.624	80.0, *P* > 0.9999
GM% IFN-γ^+^CD8^+^ T cells (95% CI)	0.050 (0.033–0.077)	0.071 (0.040–0.125)	0.059 (0.033–0.104)	0.062 (0.032–0.123)
%positive (≥ cut-off at 0.0075%)	65.0	62.1	62.5, *P* = 0.842	62.5, *P* > 0.9999
GM% IL-2^+^CD8^+^ T cells (95% CI)	0.017 (0.014–0.022)	0.041 (0.027–0.063)	0.050 (0.031–0.080)	0.066 (0.034–0.128)
%positive (≥ cut-off at 0.0075%)	58.3	65.5	68.8, *P* = 0.318	65.0, *P* > 0.9999
S-specific T-cell responses on flow cytometry				
* n*	60	59	48	41
GM% IFN-γ^+^CD4^+^ T cells (95% CI)	0.023 (0.015–0.036)	0.016 (0.010–0.027)	0.022 (0.012–0.040)	0.025 (0.013–0.048)
%positive (≥ cut-off at 0.005%)	70.0	57.6	58.3, *P* = 0.229	68.3, *P* = 0.303
GM% IL-2^+^CD4^+^ T cells (95% CI)	0.015 (0.011–0.020)	0.017 (0.010–0.027)	0.020 (0.012–0.035)	0.031 (0.017–0.059)
%positive (≥ cut-off at 0.005%)	73.3	61.0	60.4, *P* = 0.214	68.3* P* = 0.528
GM% IFN-γ^+^CD8^+^ T cells (95% CI)	0.014 (0.009–0.024)	0.017 (0.010–0.031)	0.014 (0.008–0.025)	0.010 (0.005–0.019)
%positive (≥ cut-off at 0.005%)	48.3	49.2	45.8, *P* = 0.848	39.0,* P* = 0.414
GM% IL-2^+^CD8^+^ T cells (95% CI)	0.006 (0.005–0.008)	0.009 (0.006–0.015)	0.012 (0.007–0.020)	0.010 (0.005–0.018)
%positive (≥ cut-off at 0.005%)	48.3	47.5	52.1, *P* = 0.847	41.5%, *P* = 0.683
N-specific T-cell responses on flow cytometry				
* n*	60	58	48	40
GM% IFN-γ^+^CD4^+^ T cells (95% CI)	0.011 (0.008–0.017)	0.014 (0.008–0.024)	0.022 (0.011–0.043)	0.023 (0.012–0.047)
%positive (≥ cut-off at 0.005%)	55.0	53.5	54.2, *P* > 0.9999	60.0, *P* = 0.542
GM% IL-2^+^CD4^+^ T cells (95% CI)	0.013 (0.009–0.018)	0.020 (0.012–0.033)	0.028 (0.015–0.053)	0.030 (0.015–0.059)
%positive (≥ cut-off at 0.005%)	66.7	60.3	60.4, *P* = 0.549	65.0, *P* = 0.677
GM% IFN-γ^+^CD8^+^ T cells (95% CI)	0.008 (0.005–0.012)	0.015 (0.008–0.028)	0.016 (0.008–0.031)	0.013 (0.006–0.026)
%positive (≥ cut-off at 0.005%)	31.7	41.4	43.8, *P* = 0.232	40.0, *P* > 0.9999
GM% IL-2^+^CD8^+^ T cells (95% CI)	0.0042 (0.003–0.005)	0.010 (0.006–0.016)	0.014 (0.008–0.024)	0.014 (0.007–0.027)
%positive (≥ cut-off at 0.005%)	28.3	41.4	47.9, *P* = 0.046	50.0, *P* = 0.417
M-specific T-cell responses on flow cytometry				
* n*	60	59	48	40
GM% IFN-γ^+^CD4^+^ T cells (95% CI)	0.007 (0.005–0.010)	0.006 (0.004–0.009)	0.008 (0.005–0.014)	0.010 (0.005–0.020)
%positive (≥ cut-off at 0.005%)	36.7	23.7	35.4, *P* > 0.9999	32.5, *P* = 0.365
GM% IL-2^+^CD4^+^ T cells (95% CI)	0.006 (0.004–0.007)	0.006 (0.004–0.009)	0.011 (0.006–0.019)	0.014 (0.006–0.029)
%positive (≥ cut-off at 0.005%)	46.7	25.4	41.7, *P* = 0.698	45.0, *P* = 0.052
GM% IFN-γ^+^CD8^+^ T cells (95% CI)	0.006 (0.004–0.009)	0.005 (0.003–0.008)	0.007 (0.004–0.011)	0.012 (0.006–0.0242)
%positive (≥ cut-off at 0.005%)	25.0	13.6	29.2, *P* = 0.667	42.5, *P* = 0.002
GM% IL-2^+^CD8^+^ T cells (95% CI)	0.004 (0.003–0.005)	0.004 (0.003–0.006)	0.005 (0.003–0.008)	0.012 (0.006–0.024)
%positive (≥ cut-off at 0.005%)	23.3	18.6	27.1, *P* = 0.662	47.5, *P* = 0.004

*SARS-CoV-2* severe acute respiratory syndrome coronavirus 2, *S* spike protein, *N* nucleocapsid protein, *M* membrane protein, *SNM* sum of individual S, N, and M peptide pools, *GM* geometric mean, *CI* confidence interval, *IFN-*γ interferon-gamma, *IL-2* interleukin-2. *P* values compare the proportion of positive responses between intramuscular and intradermal administration by Fisher’s exact test

**Fig. 2:**
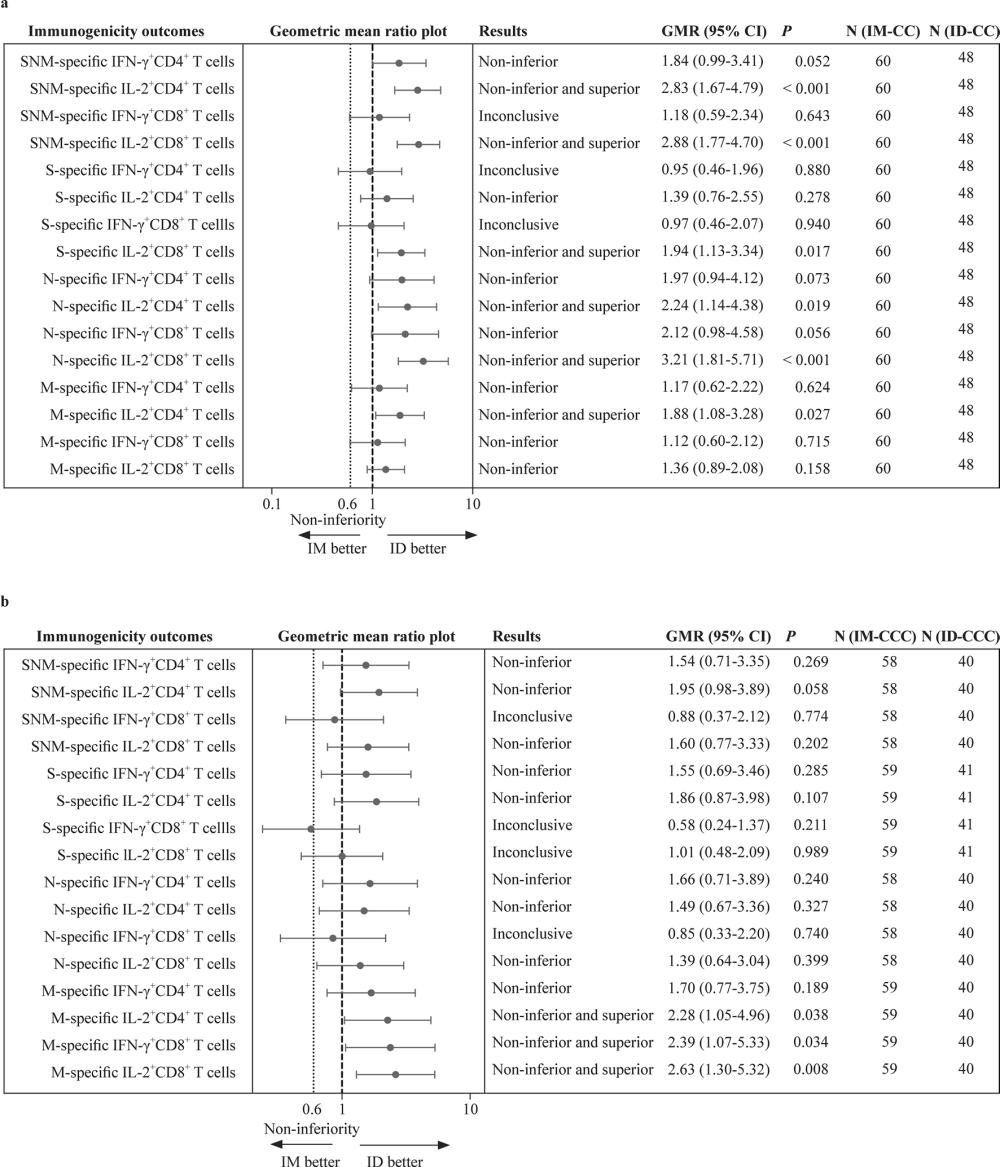
Superiority and non-inferiority hypothesis testing of cellular immunogenicity against wild-type SARS-CoV-2 post-dose 2 and post-dose 3 of vaccination in the evaluable analysis population. Adolescents receiving 2 doses of CoronaVac administered intramuscularly (IM-CC) or intradermally (ID-CC) (**a**) and 3 doses of CoronaVac administered intramuscularly (IM-CCC) or intradermally (ID-CCC) (**b**) were tested for T-cell responses by flow-cytometry-based intracellular cytokine staining assays specific to S, N and M post-dose 2 or post-dose 3. The results of SNM-specific T-cell responses were calculated from the sum of responses of the individual S, N and M peptide pools. Dots and error bars show GMR estimates and two-sided 95% CI, respectively. *GMR* geometric mean ratio, *SNM* sum of individual S, N, and M peptide pools, *S* spike protein, *N* nucleocapsid protein, *M* membrane protein, *IFN-γ* interferon-γ, *IL-2* interleukin-2, *ID* intradermal, *IM* intramuscular, *CI* confidence interval

After 2 doses of ID CoronaVac, SNM-specific IL-2^+^CD4^+^, SNM-specific IL-2^+^CD8^+^, S-specific IL-2^+^CD8^+^, N-specific IL-2^+^CD4^+^, N-specific IL-2^+^CD8^+^ and M-specific IL-2^+^CD4^+^ T-cell responses were superior and non-inferior to IM (Fig. 2a). SNM-specific IFN-γ^+^CD8^+^, S-specific IFN-γ^+^CD4^+^ and S-specific IFN-γ^+^CD8^+^ T-cell responses were inconclusive, whilst other T-cell responses were non-inferior. Additionally, evaluable ID-CCC satisfied superior and non-inferior criteria for M-specific IL-2^+^CD4^+^, IFN-γ^+^CD8^+^ and IL-2^+^CD8^+^ T-cell responses compared to IM-CCC (Fig. 2b). SNM-specific IFN-γ^+^CD8^+^, S-specific IFN-γ^+^CD8^+^, S-specific IL-2^+^CD8^+^ and N-specific IFN-γ^+^CD8^+^ T-cell responses were inconclusive, whilst the other T-cell responses were non-inferior. In general, baseline values of cellular immunogenicity outcomes were below the levels of detection and increased after each vaccine dose (Supplementary Table 4; Supplementary Fig. 4). These results were consistent with superiority and non-inferiority testing in the expanded analysis populations (Supplementary Table 5; Supplementary Fig. 5). Overall, none of the cellular immunogenicity outcomes tested for groups that received ID was inferior compared to IM.

### Longitudinal immunogenicity changes between doses over time within the same group of IM or ID and between groups

Antibody and T-cell responses were compared between post-doses 2 and 3 in the evaluable analysis populations. Overall, dose 3 induced higher humoral responses for S IgG, S-RBD IgG, sVNT, PRNT90, PRNT50, S IgG avidity and S IgG FcγRIIIa-binding than dose 2 by the IM or ID route (Supplementary. Fig. 6). Additionally, there were higher SNM-specific IL-2^+^CD4^+^, SNM-specific IL-2^+^CD8^+^ and N-specific IL-2^+^CD8^+^ T-cell responses after IM-CCC than after IM-CC (Supplementary Fig. 7). ID-CCC induced a higher M-specific IL-2^+^CD8^+^ T-cell response than ID-CC.

### Estimation of vaccine efficacies from different doses and routes of administration of CoronaVac based on neutralisation titers

**Fig. 3:**
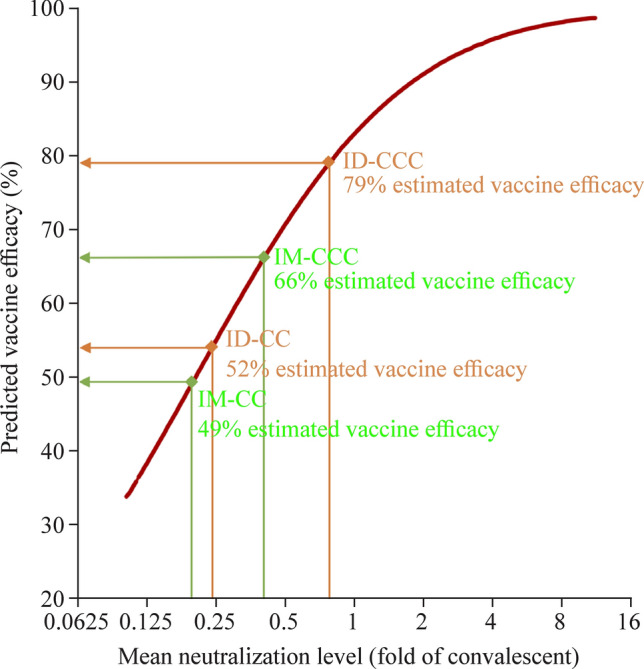
Estimation of vaccine efficacies for 2 doses and 3 doses of CoronaVac by intramuscular or intradermal administration vaccination in the evaluable analysis population based on neutralisation titers against SARS-CoV-2. The vaccine efficacy estimates were based on neutralising antibody titers (PRNT50, or plaque reduction neutralisation test based on the reciprocal of the highest serum dilution that resulted in the cutoff of > 50%) post-dose 2 or post-dose 3 of CoronaVac administered intramuscularly (IM-CC or IM-CCC) or intradermally (ID-CC or ID-CCC), as neutralising antibodies have been established as a reliable correlate of protection that can predict vaccine efficacies against symptomatic COVID-19. Dividing the geometric mean titers of PRNT50 who received vaccination by titers from 102 convalescent sera collected on days 28–59 post-onset of illness in patients aged ≥ 18 years yielded the mean neutralising levels (fold of convalescent). Extrapolation of the point estimates of the vaccine efficacies from the best fit of the logistic model was performed as previously described [14, 15, 40]. IM-CC (*n* = 119) and IM-CCC (*n* = 60), post-dose 2 and post-dose 3 of vaccine administered intramuscularly, respectively; ID-CC (*n* = 54) and ID-CCC (*n* = 42), post-dose 2 and post-dose 3 of vaccine administered intradermally, respectively. *SARS-CoV-2* severe acute respiratory syndrome coronavirus 2, *COVID-19* coronavirus disease 2019, *PRNT* plaque reduction neutralisation titre, *ID* intradermal, *IM* intramuscular

Levels of neutralising antibodies have been regarded as a correlate of protection. Hence, we extrapolated our PRNT50 results from evaluable adolescents who received 2 or 3 doses of IM or ID CoronaVac with vaccine efficacies against symptomatic COVID-19 by normalisation to convalescent sera, as previously described [14, 40]. The mean neutralisation levels of IM-CC, ID-CC, IM-CCC and ID-CCC were 0.19, 0.22, 0.40 and 0.79, corresponding to 49%, 52%, 66% and 79% vaccine efficacies, respectively (Fig. 3). Using PRNT90 instead of PRNT50 yielded similar findings (data not shown).

### Omicron-specific humoral and cellular immunogenicity post-dose 2 and post-dose 3 of ID CoronaVac

**Fig. 4:**
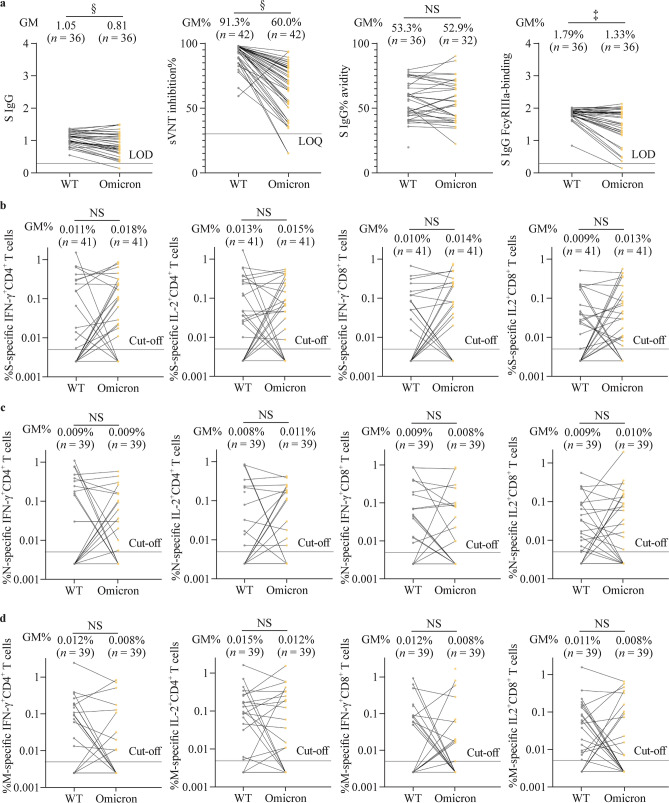
Omicron variant-specific humoral and cellular immunogenicity post-dose 3 of CoronaVac administered intradermally in the evaluable analysis population. Humoral (**a**) and (**b**–**d**) cellular immunogenicity outcomes against WT SARS-CoV-2 and the Omicron variant post-dose 3 of CoronaVac administered intradermally. Data labels and centre lines show GM estimates, and error bars show 95% CI. *P* values were derived from two-tailed unpaired *t* test after natural logarithmic transformation. *SARS-CoV-2* severe acute respiratory syndrome coronavirus 2, *GM* geometric mean, *WT* wild type, *S* spike protein, *N* nucleocapsid protein, *M* membrane protein, *IgG* immunoglobulin G, *sVNT* surrogate virus neutralisation test, *FcγRIIIa* Fcγ receptor IIIa, *LOD* limit of detection, *IFN-γ* interferon-γ, *IL-2* interleukin-2, *CI* confidence interval, *NS* no significant difference. ^‡^*P* < 0.001; ^§^*P* < 0.0001

For ID-CCC, humoral responses tested against the Omicron variant were significantly lower than those against WT SARS-CoV-2 for S IgG, sVNT inhibition and S IgG FcγRIIIa-binding (Fig. 4a). S IgG avidity and all the cellular immunogenicity outcomes were similar between Omicron and WT (Fig. 4a–d).

### Reactogenicity and safety of IM or ID CoronaVac

**Fig. 5:**
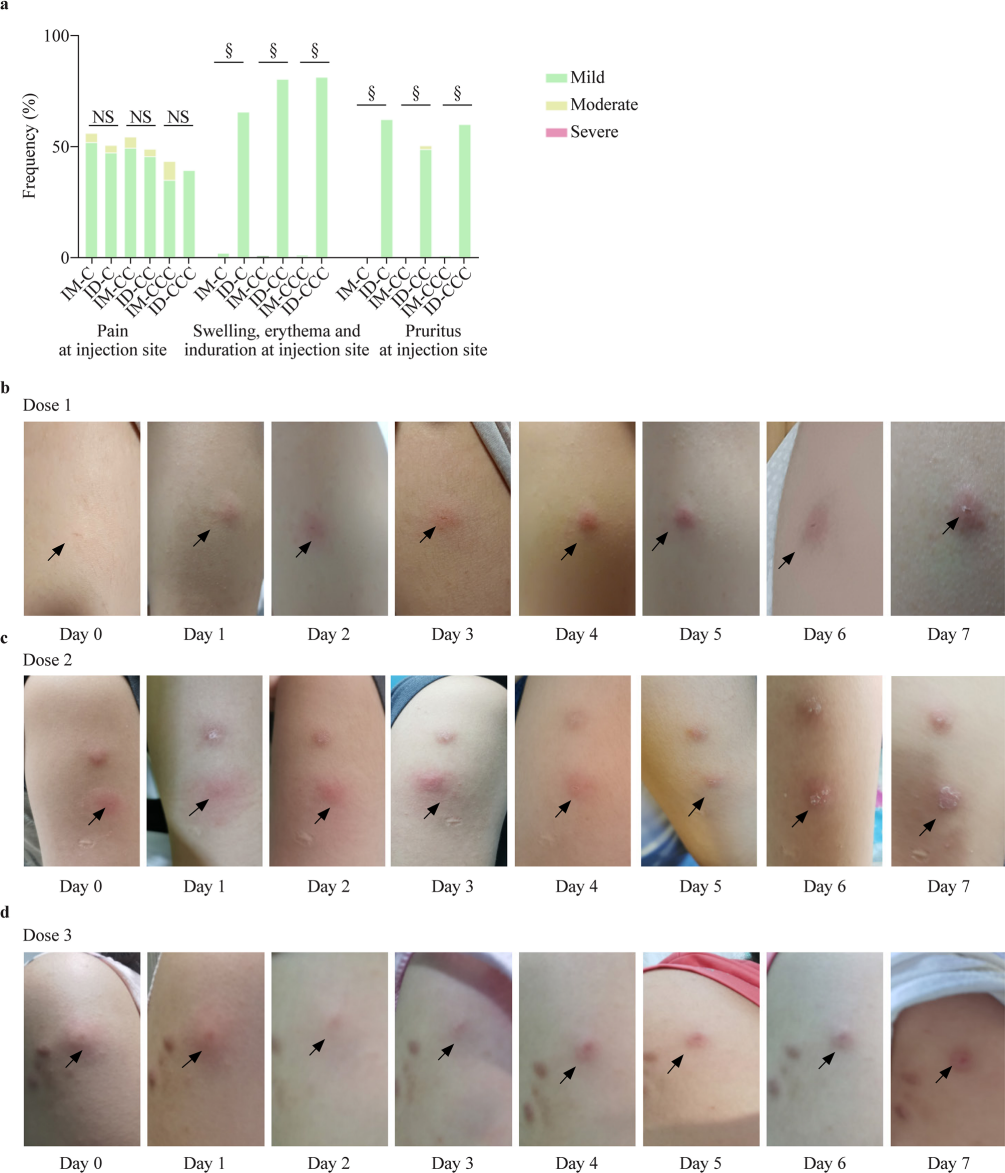
Local adverse reactions in the healthy safety population after doses 1, 2 and 3 of CoronaVac by intramuscular or intradermal administration. **a** Local adverse reactions 7 days after each dose of CoronaVac administered by intramuscular or intradermal injections were solicited from participants in the healthy safety population. Data are shown as percentages of the respective adverse reaction of any severity; **b**–**d** photos are representative of the typical injection site reactions manifested for 7 days after doses 1 (**b**), 2 (**c**) or 3 (**d**) of CoronaVac administered intradermally. IM-CC (*n* = 119) and IM-CCC (*n* = 94), post-dose 2 and post-dose 3 of vaccine administered intramuscularly, respectively; ID-CC (*n* = 59) and ID-CCC (*n* = 45), post-dose 2 and post-dose 3 of vaccine administered intradermally, respectively. *ID* intradermal, *IM* intramuscular, *NS* no significant difference. ^§^*P* < 0.0001

**Fig. 6:**
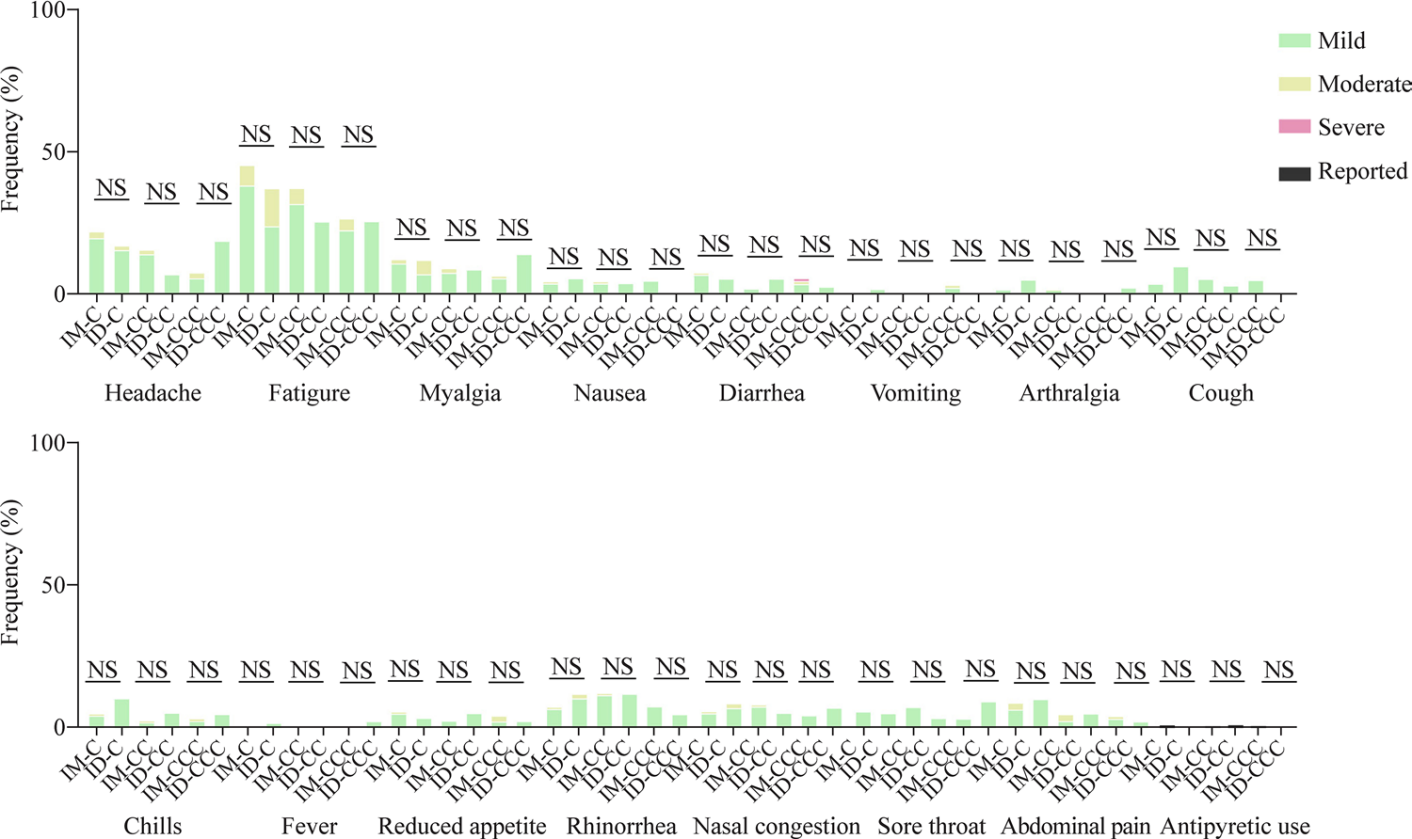
Systemic adverse reactions in the healthy safety population after doses 1, 2 and 3 of CoronaVac by intramuscular or intradermal administration. Adverse reactions 7 days after each dose of CoronaVac administered by intramuscular or intradermal injections were solicited from participants in the healthy safety population. Data are shown as percentages of the respective adverse reaction of any severity. IM-CC (*n* = 119) and IM-CCC (*n* = 94), post-dose 2 and post-dose 3 of vaccine administered intramuscularly, respectively; ID-CC (*n* = 59) and ID-CCC (*n* = 45), post-dose 2 and post-dose 3 of vaccine administered intradermally, respectively. *ID* intradermal, *IM* intramuscular, *NS* no significant difference

In the healthy safety population, pain at the injection site was the most reported AR for IM, which was similar to ID (Fig. 5a) pruritus, at the injection site than IM. Most recipients developed these symptoms within minutes after ID, which progressed locally at the site of inoculation over 1–2 weeks and subsided over several weeks (Fig. 5b–d; Supplementary Fig. 8). Systemic ARs were similar between IM and ID (Fig. 6). There were 13 AEs reported within 28 days after vaccination (8 for IM and 5 for ID) and no SAEs for either administration route (Supplementary Table 6).

## Discussion

This is the first study to assess the immunogenicity, reactogenicity and safety of ID administration of an inactivated COVID-19 vaccine at a full dose, which demonstrated superior antibody responses and T-cell responses against the SARS-CoV-2 M protein in adolescents who received three injections. ID-CCC elicited higher antibody responses across all parameters tested and M-specific IL-2^+^CD8^+^ T-cell responses than ID-CC. These data estimated the vaccine efficacy of ID-CCC to be 79%, which was about 30% higher than the 2 doses of IM observed in this study (49%), our recent immuno-bridging publication (50%) and CoronaVac’s initial study in Brazil (51%) [7, 14, 15]. Whether the observed superior immunogenicity and estimated efficacy from ID translate to actual higher clinical efficacy and effectiveness needs to be further studied. This cohort remains in our ongoing study and is scheduled for follow-up visits over three years, which will provide the opportunity to trace the clinical protection conferred by ID compared to IM.

The long-term immune protection from different types and routes of vaccination, particularly against VOCs, is incompletely established. In this study, the levels of anti-spike antibodies and their FcγRIIIa-binding against Omicron were lower than those against WT after ID-CCC. However, S IgG avidity and cellular immunogenicity against Omicron were maintained, which can possibly explain the persistently high real-life vaccine effectiveness even when it is known that there is waning of quantitative serum antibody concentrations against VOCs [5, 12]. Indeed, several human studies have shown that T-cell immunity can persist for years after prior exposure and mitigate disease severity when neutralising antibodies are reduced, and pre-existing antigen-specific T cells are protective against influenza viral infections, severity of symptoms and viral shedding [41–45]. In addition, vaccine-induced T-cell responses against the highly conserved structural SARS-CoV-2 M protein confer partial protection from lung pathology in a murine model [46].

Interestingly, S IgG avidity for ID-CC was inferior to that for IM-CC. However, this was reversed after 3 doses. This was accompanied by a simultaneous shift from non-inferior to superior neutralising antibodies and sVNT, whilst the differences in S IgG and S-RBD IgG between IM and ID became less pronounced. We speculate that despite reduced differences in the quantitative anti-S IgG concentrations after dose 3 between ID and IM, there was greater antibody function and quality from the higher avidity that correspondingly enhanced the neutralisation of SARS-CoV-2 [47]. The high-affinity antibodies that resulted in higher avidity binding are indicative of class switching in germinal centres, affinity maturation and longer-lasting functional antibody responses. Furthermore, FcγRIIIa functions were also increased by ID vaccination. Our recent publication on BNT162b2 by IM demonstrated consistent increases in S IgG avidity across time, whilst the concentrations of IgGs waned before dose 3 was administered as a booster vaccination to adolescents [33]. These findings support the notion that effective vaccination induces efficient immunity characterised by antibodies that have high avidity and increased effector functions against SARS-CoV-2 rather than large quantities of low-quality immunoglobulins [48]. In this regard, S IgG avidity maturation in our cohort appears to be markedly boosted by dose 3 of ID CoronaVac, and the kinetics of this antibody response are significantly different from IM.

This study had some limitations. It was not possible to blind the participants because receipt of IM or ID injections was readily differentiable, and the technique of administration for the two routes was also noticeably distinct to the clinical staff. It can be viewed that this unblinded, non-randomised study design is a limitation and has the potential for selection bias. However, since the age, sex and ethnic distributions were similar between groups, the immunogenicity comparisons should be valid. Importantly, this practical approach can be directly interpretable for real-life applicability. For example, although the immunogenicity of ID appears greater than that of IM, ID injections were associated with more local reactions. This known adverse effect can be a reason for some individuals to select IM rather than ID in the real world. Although ARs were predominantly local symptoms and no SAEs occurred, participants who received ID endured weeks of induration and itching at the site of injection, most of whom were described as tolerable. These findings were consistent with a recent study on the novel Mpox by ID route that was associated with acceptable local reactions, as both CoronaVac and 3rd generation Mpox vaccines are incapable of replication [49]. Larger pharmacovigilance studies over longer periods of time will be required to delineate whether there are higher risks of rarer adverse effects from full-dose, intradermally administered inactivated COVID-19 vaccination. Additionally, the strict social distancing policies that the Hong Kong Government mandated during the COVID-19 period kept infection numbers low so that it was not possible to investigate vaccine efficacy in this cohort during this study period, from April 2021 to August 2022 [1, 5, 12]. On the other hand, this provided the opportunity to determine ID vaccine immunogenicity without the confounding effects of existing immunity acquired from past infections [1, 5, 12]. Moreover, we were able to estimate actual vaccine efficacies using neutralising antibodies [14, 15, 40]. Comprehensive, validated assays of antibody levels, avidity and binding and T-cell responses were a major strength of this study, yet follow-up research on clinical efficacy and real-life effectiveness against COVID-19 from ID is needed to further support the current findings. The sample size for the ID group was slightly smaller than the initial target due to the unforeseeable wave of Omicron infections that swept across Hong Kong during the study period in which doses 2 and 3 were administered. This led to the exclusion of more enrolled participants than expected, whilst a few others under quarantine as close contacts could not provide blood samples within the evaluable window, which can lead to selection bias. Despite the reduced sample size, detection of significant superiority in the ID group was still achieved in many of the major immunogenicity outcomes. The larger sample size from the expanded analysis population confirmed the observed superior immunogenicity. PRNT against Omicron was not performed, as this was not a pre-specified primary outcome, and this variant had not emerged by the time of study design. Nevertheless, sVNT results against the Omicron variant were added later and are available. sVNT has been a reliable surrogate to evaluate antibody neutralisation. This study focussed on adolescents, and thus, the immunogenicity of ID CoronaVac will need to be explored for adults and young children as well.

The specific populations that would likely benefit most from this enhanced immunogenicity of ID CoronaVac are the unvaccinated and high priority group that includes immunocompromised patients, young individuals with comorbidities, elderly individuals, pregnant persons, and frontline health workers. This aligns with the World Health Organization’s most recent recommendation on 28 March 2023 that this high priority group should receive an additional COVID-19 booster vaccination 6–12 months after the last dose. Many older adults, young children or those with debilitating chronic diseases are hesitant towards receiving novel monovalent or bivalent mRNA vaccines due to systemic adverse effects, risks of myocarditis and potentially higher association with ischaemic stroke [14, 50–52]. As this study demonstrated more frequent but tolerable local adverse effects only, it is possible that these individuals would be more accepting of ID inactivated vaccines. Future studies comparing the immunogenicity with mRNA vaccines would be worthwhile and if shown to be similar to ID CoronaVac, then ID can offer a cost-effective option for areas with limited access to the more immunogenic mRNA vaccines that have higher financial or storage demands. A previous study revealed enhanced immunogenicity from ID against the hepatitis B virus in dialysis patients who was more striking 52 weeks after ID than IM [53]. Whether such durability of heightened immunogenicity in healthy individuals is conferred by ID CoronaVac against SARS-CoV-2 and novel VOCs in the future is not yet certain, which we will track in these adolescents for the next three years. Importantly, this study serves as a proof of concept and the basis for further research on ID using full doses of vaccines, rather than fractional dosing, to optimise protection against COVID-19 and other infectious diseases, particularly for those at risk of vaccine failures.

### Supplementary Information

Below is the link to the electronic supplementary material.

Supplementary file 1 (PDF 204 KB)

Supplementary file 2 (PDF 2109 KB)

Supplementary file 3 (PDF 3597 KB)

Supplementary file 4 (PDF 157 KB)

## Data Availability

The study’s protocol and the statistical analysis plan are included in the supplementary information. To maintain confidentiality for the participants, only de-identified data shall be shared with scientific investigators who submit a justifiable inquiry to lauylung@hku.hk. The study is ongoing, and therefore data will be available upon request one month after the completion of the study in 2025.
