# Supplementary material for: Superior antibody and membrane protein-specific T-cell responses to CoronaVac by intradermal versus intramuscular routes in adolescents
Source: World J Pediatr. 2023 Dec 12;20(4):353–70. doi: 10.1007/s12519-023-00764-0 (PMC11052846; doi:10.1007/s12519-023-00764-0)
Supplement: Supplementary file 3 — (PDF 3597 KB) [file 12519_2023_764_MOESM3_ESM.pdf]

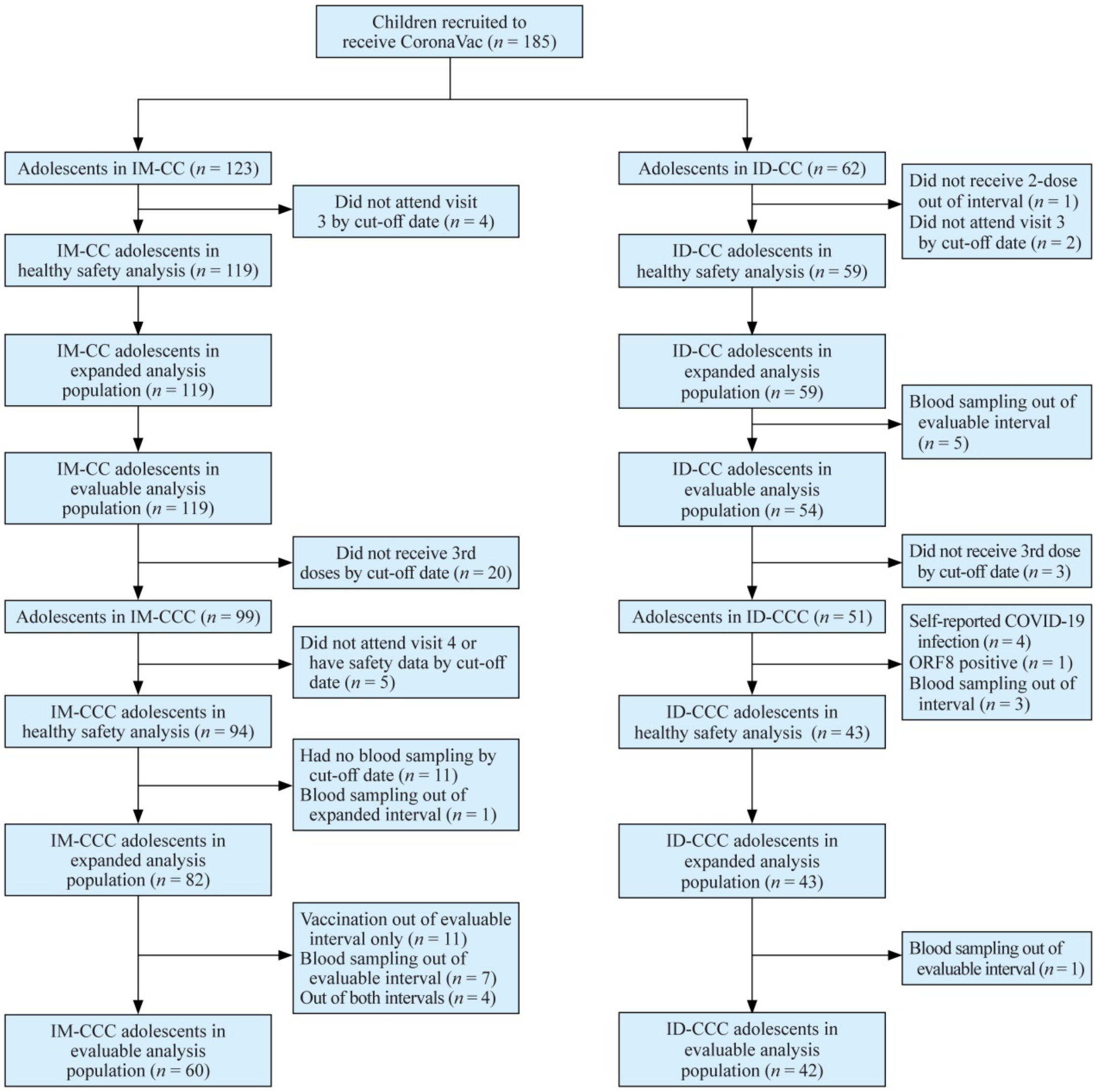


**Supplementary Fig. 1** Study flow diagram. The evaluable analysis population comprised of uninfected participants without major protocol deviations and valid results. For confirmation of results, an expanded analysis population with more relaxed time windows between doses and blood sample were included and analyzed additionally. IM-CC and IM-CCC, 2 and 3 doses of vaccine administered intramuscularly, respectively; ID-CC and ID-CCC, 2 and 3 doses of vaccine administered intradermally, respectively; ORF8 IgG (a serological marker of past natural SARS-CoV-2 infection). *ID* intradermal, *IM* intramuscular, *SARS-CoV-2* severe acute respiratory syndrome coronavirus 2, *COVID-19* coronavirus disease 2019, *ORF8* open reading frame 8, *IgG* immunoglobulin G

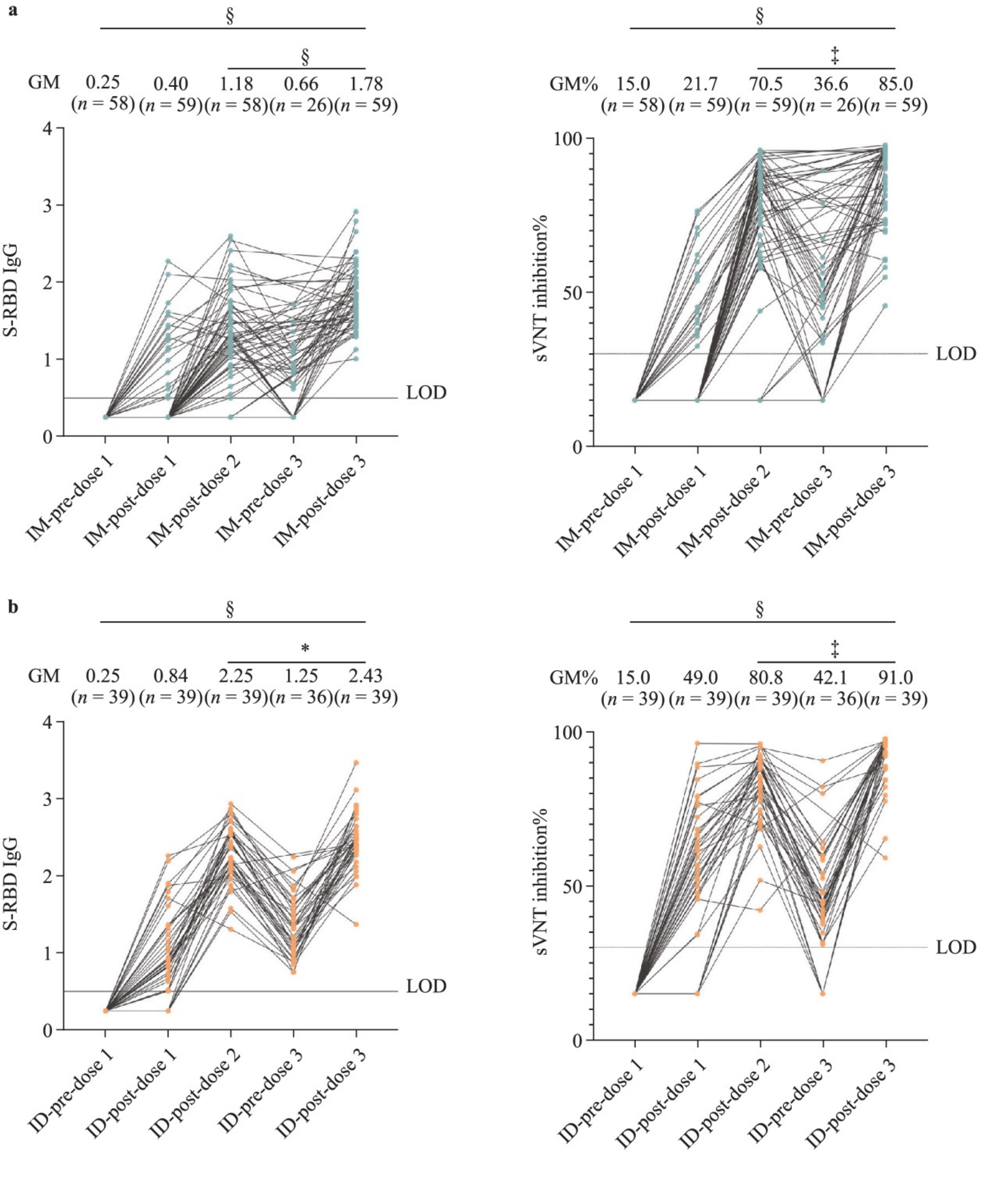


**Supplementary Fig. 2** Baseline (pre-dose 1) values and longitudinal antibody responses over time against wild type SARS-CoV-2 in individual adolescents. Adolescents receiving (**a**) intramuscular or (**b**) intradermal injections of CoronaVac were tested for humoral immunogenicity outcomes. Dots show GM estimates. Pre-dose 1 is equivalent to baseline; IM-post-dose 2 is equivalent to IM-CC; IM-post-dose 3 is equivalent to IM-CCC; ID-post-dose 2 is equivalent to ID-CC; ID-post-dose 3 is equivalent to ID-CCC. *SARS-CoV-2* severe acute respiratory syndrome coronavirus 2, *ID* intradermal, *IM* intramuscular, *GM* geometric mean, *S* spike protein, *RBD* receptor-binding domain, *IgG* immunoglobulin G, *sVNT* surrogate virus neutralization test. $^{*}P < 0.05$; $^{‡}P < 0.001$; $^{§}P < 0.0001$

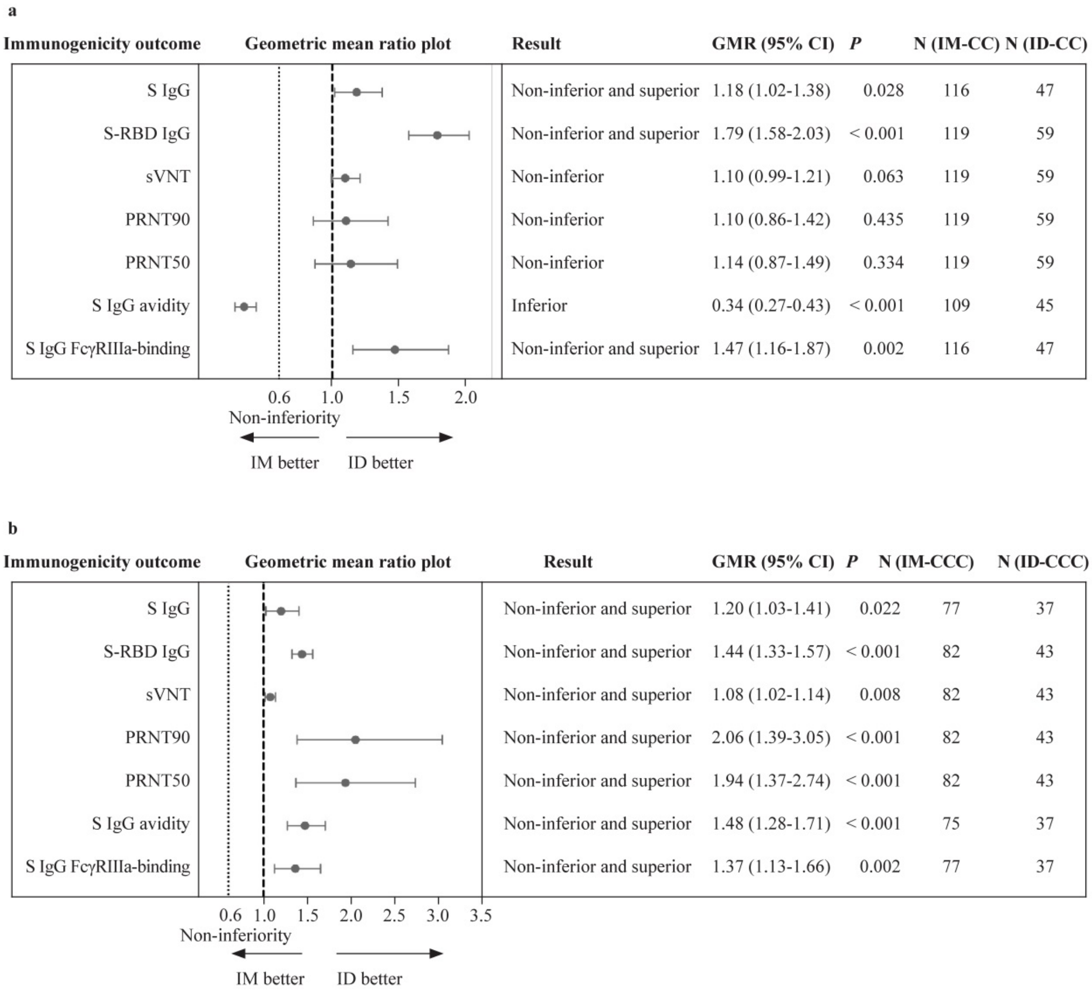


**Supplementary Fig. 3** Superiority and non-inferiority hypotheses testing of humoral immunogenicity against wild type SARS-CoV-2 post-dose 2 and post-dose 3 of vaccination the in expanded analysis population. Adolescents receiving (**a**) 2 doses of CoronaVac administered intramuscularly or intradermally and (**b**) 3 doses of CoronaVac administered intramuscularly or intradermally were tested for humoral immunogenicity outcomes in the expanded analysis population for confirmation of the findings from the evaluable analysis population. Dots and error bars show GMR estimates and two-sided 95% CI, respectively. *SARS-CoV-2* severe acute respiratory syndrome coronavirus 2, *GMR* geometric mean ratio, *S* spike protein, *N* nucleocapsid protein, *RBD* receptor-binding domain, *IgG* immunoglobulin G, *ID* intradermal, *IM* intramuscular, *sVNT* surrogate virus neutralization test, *PRNT* plaque reduction neutralization titer, *FcγRIIIa* Fcγ receptor IIIa, *CI* confidence interval

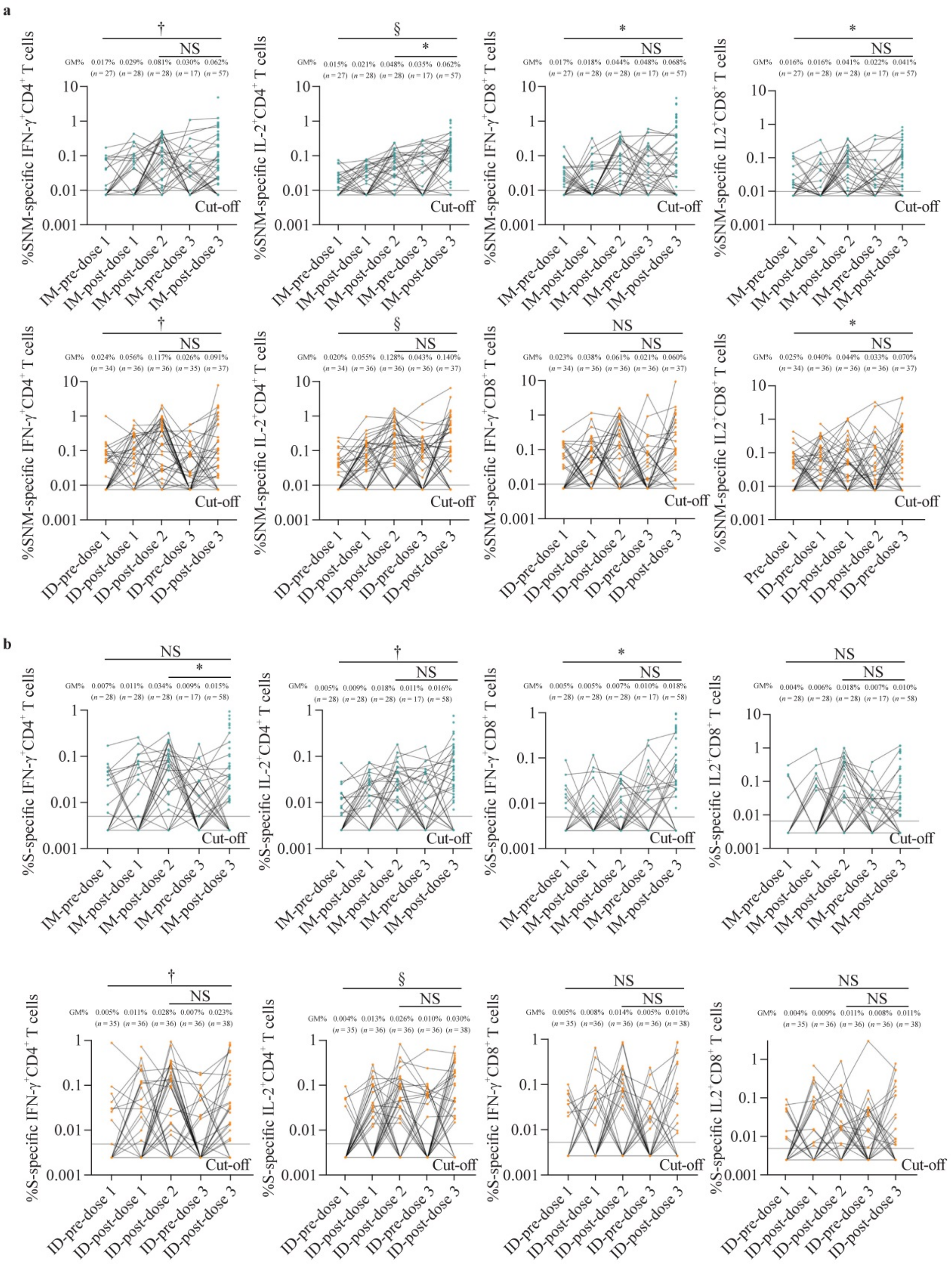

a
†
NS
GM% 0.017% 0.029% 0.081% 0.030% 0.062%
(n = 27) (n = 28) (n = 28) (n = 17) (n = 57)
%SNM-specific IFN-γ+CD4+ T cells
Cut-off
IM-pre-dose 1
IM-post-dose 1
IM-post-dose 2
IM-pre-dose 3
IM-post-dose 3
§
*
GM% 0.015% 0.021% 0.048% 0.035% 0.062%
(n = 27) (n = 28) (n = 28) (n = 17) (n = 57)
%SNM-specific IL-2+CD4+ T cells
*
NS
GM% 0.017% 0.018% 0.044% 0.048% 0.068%
(n = 27) (n = 28) (n = 28) (n = 17) (n = 57)
%SNM-specific IFN-γ+CD8+ T cells
*
NS
GM% 0.016% 0.016% 0.041% 0.022% 0.041%
(n = 27) (n = 28) (n = 28) (n = 17) (n = 57)
%SNM-specific IL2+CD8+ T cells
†
NS
GM% 0.024% 0.056% 0.117% 0.026% 0.091%
(n = 34) (n = 36) (n = 36) (n = 35) (n = 37)
%SNM-specific IFN-γ+CD4+ T cells
ID-pre-dose 1
ID-post-dose 1
ID-post-dose 2
ID-pre-dose 3
ID-post-dose 3
§
NS
GM% 0.020% 0.055% 0.128% 0.043% 0.140%
(n = 34) (n = 36) (n = 36) (n = 36) (n = 37)
%SNM-specific IL-2+CD4+ T cells
NS
NS
GM% 0.023% 0.038% 0.061% 0.021% 0.060%
(n = 34) (n = 36) (n = 36) (n = 36) (n = 37)
%SNM-specific IFN-γ+CD8+ T cells
*
NS
GM% 0.025% 0.040% 0.044% 0.033% 0.070%
(n = 34) (n = 36) (n = 36) (n = 36) (n = 37)
%SNM-specific IL2+CD8+ T cells
Pre-dose 1
ID-pre-dose 1
ID-post-dose 1
ID-post-dose 2
ID-pre-dose 3
b
NS
*
GM% 0.007% 0.011% 0.034% 0.009% 0.015%
(n = 28) (n = 28) (n = 28) (n = 17) (n = 58)
%S-specific IFN-γ+CD4+ T cells
†
NS
GM% 0.005% 0.009% 0.018% 0.011% 0.016%
(n = 28) (n = 28) (n = 28) (n = 17) (n = 58)
%S-specific IL-2+CD4+ T cells
*
NS
GM% 0.005% 0.005% 0.007% 0.010% 0.018%
(n = 28) (n = 28) (n = 28) (n = 17) (n = 58)
%S-specific IFN-γ+CD8+ T cells
NS
NS
GM% 0.004% 0.006% 0.018% 0.007% 0.010%
(n = 28) (n = 28) (n = 28) (n = 17) (n = 58)
%S-specific IL2+CD8+ T cells
†
NS
GM% 0.005% 0.011% 0.028% 0.007% 0.023%
(n = 35) (n = 36) (n = 36) (n = 36) (n = 38)
%S-specific IFN-γ+CD4+ T cells
§
NS
GM% 0.004% 0.013% 0.026% 0.010% 0.030%
(n = 35) (n = 36) (n = 36) (n = 36) (n = 38)
%S-specific IL-2+CD4+ T cells
NS
NS
GM% 0.005% 0.008% 0.014% 0.005% 0.010%
(n = 35) (n=36) (n = 36) (n = 36) (n = 38)
%S-specific IFN-γ+CD8+ T cells
NS
NS
GM% 0.004% 0.009% 0.011% 0.008% 0.011%
(n = 35) (n = 36) (n = 36) (n = 36) (n = 38)
%S-specific IL2+CD8+ T cells

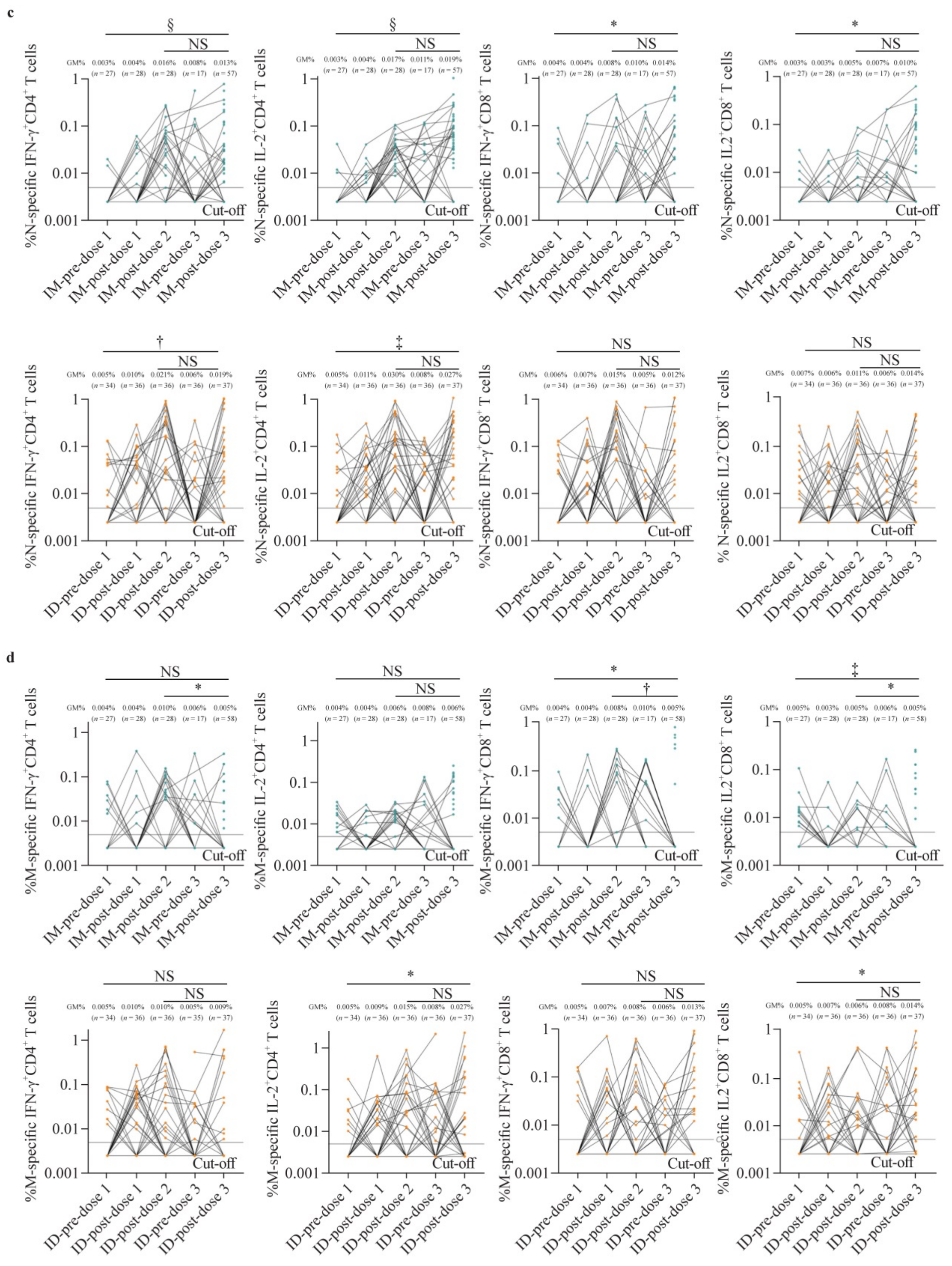


**Supplementary Fig. 4** Baseline values and longitudinal T-cell responses over time against wild type SARS-CoV-2 in individual adolescents. T-cell responses by flow-cytometry-based intracellular cytokine staining assays specific to the combined peptide pools (**a**) of S (**b**), N (**c**) and M (**d**) were measured for adolescents receiving (upper rows) intramuscular or (lower-rows) intradermal injections of CoronaVac. The results of SNM-specific T-cell responses were calculated from the sum of responses of the individual S, N and M peptide pools. Dots show GM estimates. Pre-dose 1 is equivalent to baseline; IM-post-dose 2 is

equivalent to IM-CC; IM-post-dose 3 is equivalent to IM-CCC; ID-post-dose 2 is equivalent to ID-CC; ID-post-dose 3 is equivalent to ID-CCC. *SARS-CoV-2* severe acute respiratory syndrome coronavirus 2, *S* spike protein, *N* nucleocapsid protein, *M* membrane protein, *SNM* sum of individual S, N, and M peptide pools, *IFN-γ* interferon-γ, *IL-2* interleukin-2, *ID* intradermal, *IM* intramuscular, *GM* geometric mean, *NS* no significant difference. [*]$P < 0.05$; [†]$P < 0.01$, [‡]$P < 0.001$; [§]$P < 0.0001$

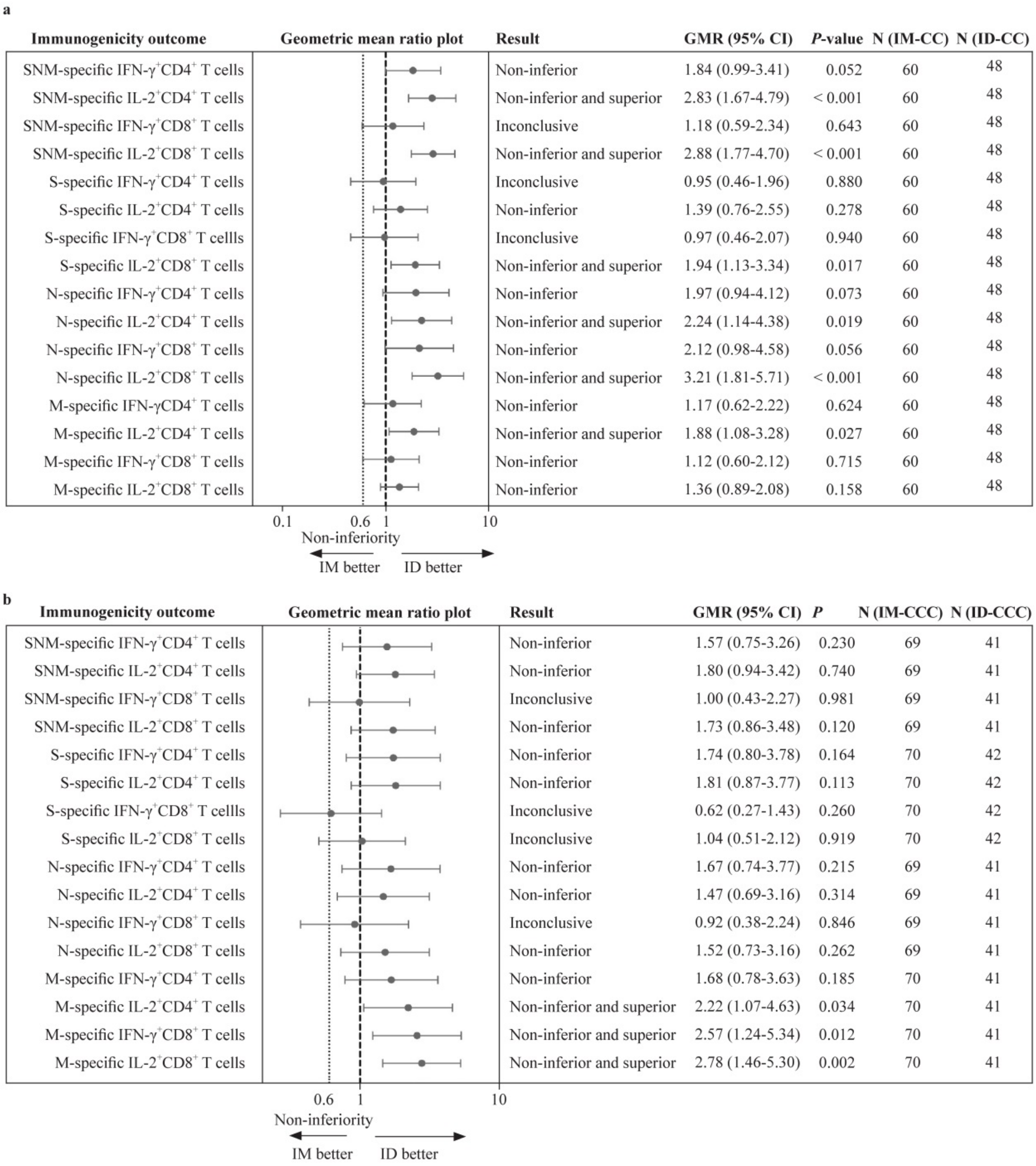


**Supplementary Fig. 5** Superiority and non-inferiority hypotheses testing of cellular immunogenicity against wild type SARS-CoV-2 post-dose 2 and post-dose 3 of vaccination in the expanded analysis population. Adolescents receiving (**a**) 2 doses of CoronaVac administered intramuscularly or intradermally and (**b**) 3 doses of CoronaVac administered intramuscularly or intradermally were tested for T-cell responses by flowcytometry-based intracellular cytokine staining assays specific to S, N and M post-dose 2 or post-dose 3 in the expanded analysis population for confirmation of the findings from the evaluable analysis population. The results of SNM-specific T-cell responses were calculated from the sum of responses of the individual S, N and M peptide pools. Dots and error bars show GMR estimates and two-sided 95% CI, respectively. *SARS-CoV-2* severe acute respiratory syndrome coronavirus 2, *GMR* geometric mean ratio, *IFN-γ* interferon-γ, *IL-2* interleukin-2, *S* spike protein, *N* nucleocapsid protein, *M* membrane protein, *SNM* sum of individual S, N, and M peptide pools, *ID* intradermal, *IM* intramuscular, *CI* confidence interval

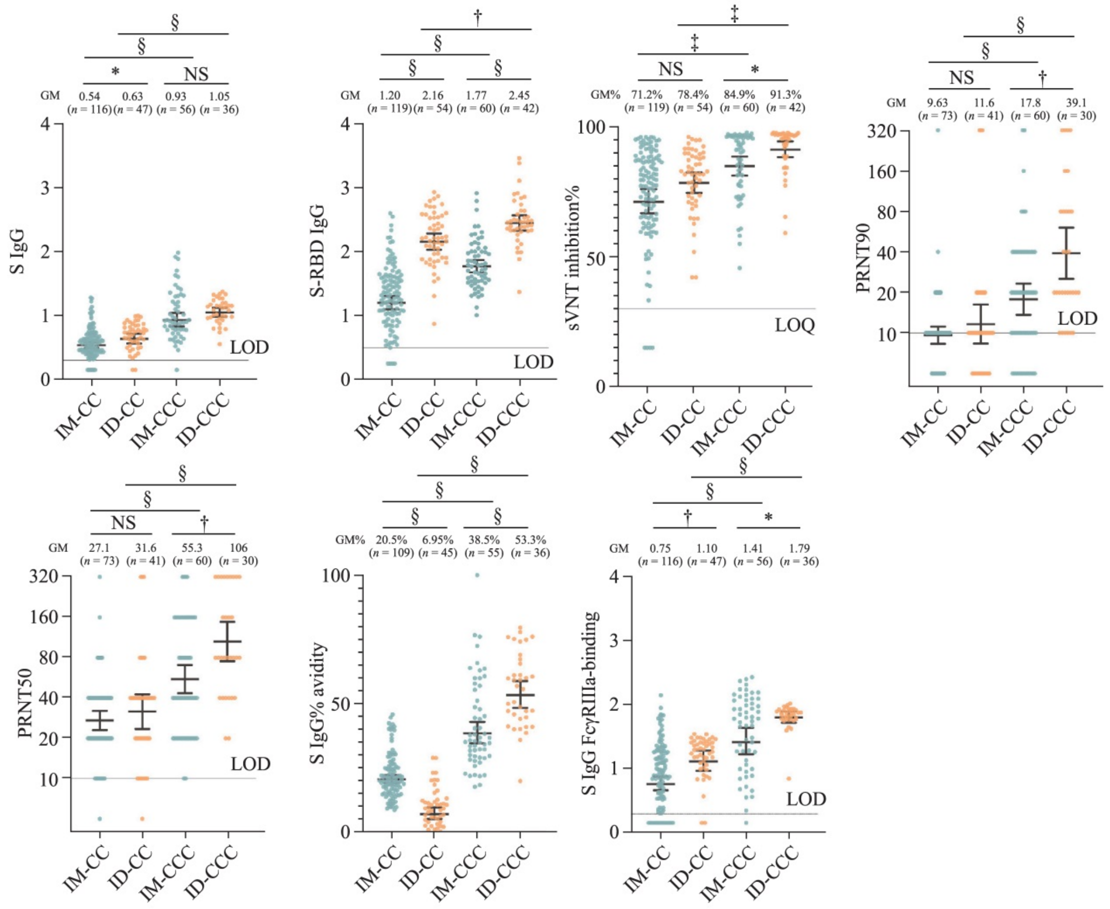


**Supplementary Fig. 6** Changes in antibody responses post-dose 2 and post-dose 3 of vaccination in the evaluable analysis population. Humoral immunogenicity outcomes were compared between CoronaVac administered intramuscularly (IM) or intradermally (ID) post-dose 2 and post-dose 3. Data labels and center lines show GM estimates, and error bars show 95% CI. *P* values were derived from two-tailed unpaired t test after natural logarithmic transformation. IM-CC and IM-CCC: 2 and 3 doses of vaccine administered intramuscularly, respectively; ID-CC and ID-CCC: 2 and 3 doses of vaccine administered intradermally, respectively. *GM* geometric mean, *S* spike protein, *IgG* immunoglobulin G, *RBD* receptor-binding domain, *sVNT* surrogate virus neutralization test, *PRNT* plaque reduction neutralization titer, *FcγRIIIa* Fcγ receptor IIIa, *LOD* limit of detection, *CI* confidence interval, *NS* no significant difference. $^{*}P < 0.05$; $^{\dagger}P < 0.01$, $^{\ddagger}P < 0.001$; $^{\S}P < 0.0001$

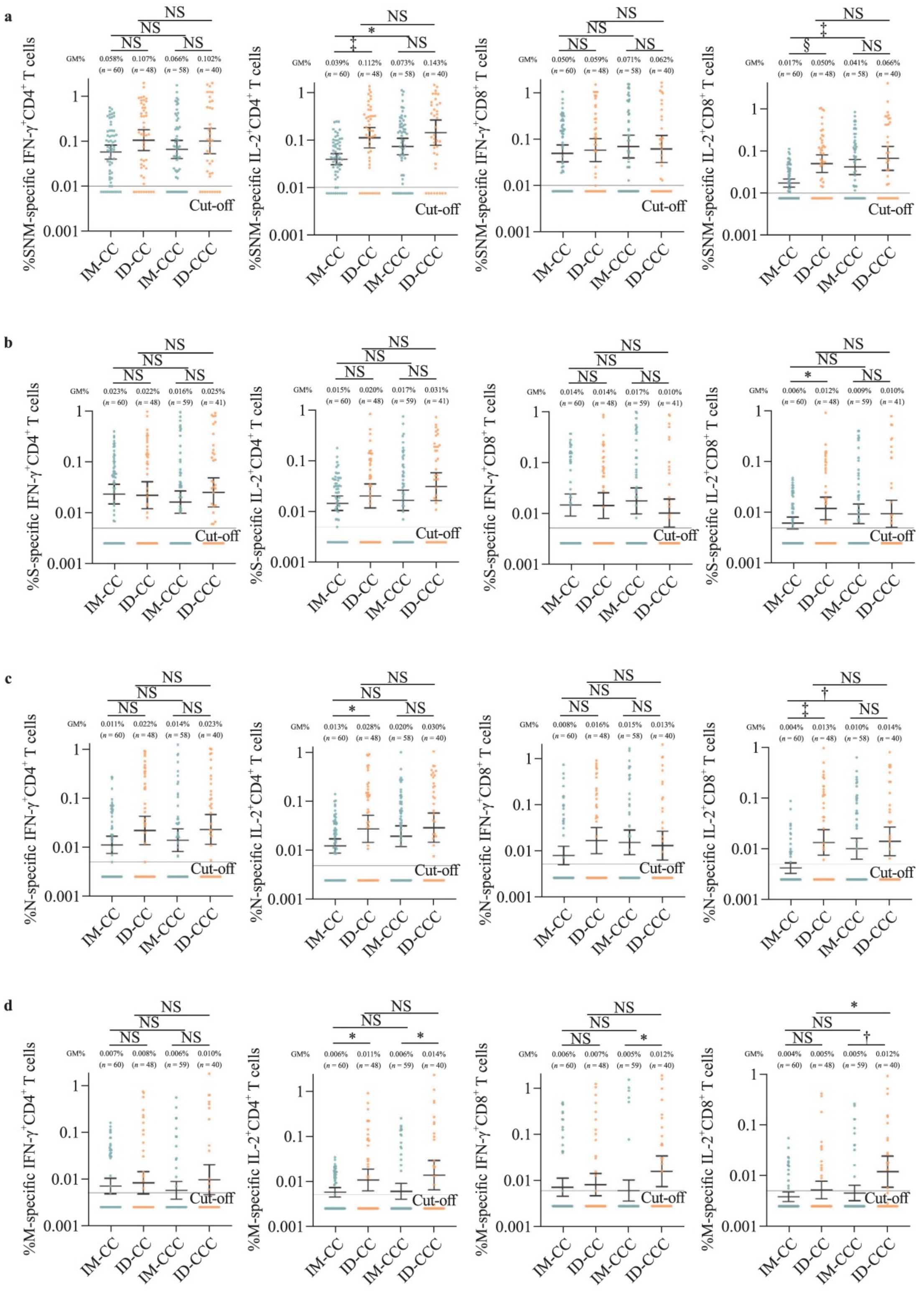


**Supplementary Fig. 7** Changes in T-cell responses post-dose 2 and post-dose 3 of vaccination in the evaluable analysis population. Cellular immunogenicity outcomes were compared between CoronaVac administered intramuscularly (IM) or intradermally (ID) post-dose 2 and post-dose 3. The results of SNM-specific T-cell responses were calculated from the sum of responses of the individual S, N and M peptide pools. Data labels and center lines show GM

estimates, and error bars show 95% CI. *P* values were derived from two-tailed unpaired *t* test after natural logarithmic transformation. IM-CC and IM-CCC: 2 and 3 doses of vaccine administered intramuscularly, respectively; ID-CC and ID-CCC: 2 and 3 doses of vaccine administered intradermally, respectively. *GM* geometric mean, *S* spike protein, *N* nucleocapsid protein, *M* membrane protein, *SNM* sum of individual S, N, and M peptide pools, *IFN-γ* interferon-γ, *IL-2* interleukin-2, *CI* confidence interval, *NS* no significant difference. $^{*}P < 0.05$; $^{\dagger}P < 0.01$, $^{\ddagger}P < 0.001$; $^{\S}P < 0.0001$

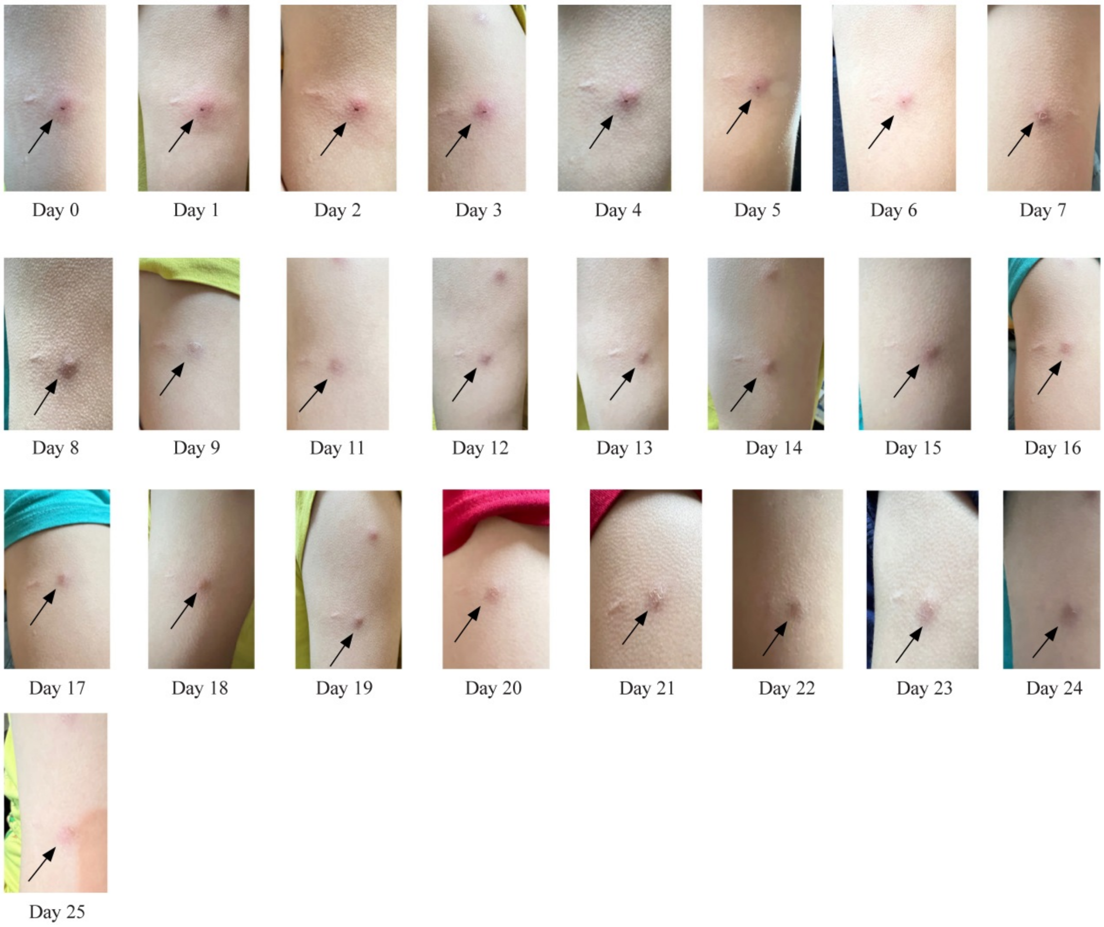


**Supplementary Fig. 8** The evolution of cutaneous manifestations at the site of inoculation after dose 2 of intradermal CoronaVac. The photos from an adolescent participant are representative of the typical injection site manifestations days 0 to 25 after dose 2 of CoronaVac administered intradermally.
