# Supplementary material for: Superior antibody and membrane protein-specific T-cell responses to CoronaVac by intradermal versus intramuscular routes in adolescents
Source: World J Pediatr. 2023 Dec 12;20(4):353–70. doi: 10.1007/s12519-023-00764-0 (PMC11052846; doi:10.1007/s12519-023-00764-0)
Supplement: Supplementary file 4 — (PDF 157 KB) [file 12519_2023_764_MOESM4_ESM.pdf]

**Supplementary Table 1.** Characteristics of the study participants in the healthy safety population	
**Intradermal**	
Numbers of participants	59
Male sex	30 (50.8)
Han Chinese	59 (100.0)
Age (y)	13.7 (1.5)
**Intramuscular**	
Number of participants	119
Male sex	65 (54.6)
Han Chinese	119 (100.0)
Age (y)	14.3 (17.6)
Values are either counts (%) or means (standard deviations)	

Supplementary Table 2. Humoral immunogenicity outcomes against wild type SARS-CoV-2 at baseline in the evaluable and expanded analysis populations

Variables	Intramuscular	Intradermal	*P*
	Pre-dose 1	Pre-dose 1	
**S-RBD IgG on ELISA (evaluable)**			
N	119	54	
GM OD450 value (95% CI)	0.250 (0.250-0.250)	0.250 (0.250-0.250)	NA
%positive (≥ LOD at 0.3)	0.0	0.0	> 0.9999
**S-RBD ACE2-blocking antibody on sVNT (evaluable)**			
N	119	54	
GM% inhibition (95% CI)	15.0 (15.0-15.0)	15.0 (15.0-15.0)	NA
%positive (≥ LOQ at 30%)	0.0	0.0	> 0.9999
**S-RBD IgG on ELISA (expanded)**			
N	119	59	
GM OD450 value (95% CI)	0.250 (0.250-0.250)	0.250 (0.250-0.250)	NA
%positive (≥ LOD at 0.3)	0.0	0.0	> 0.9999
**S-RBD ACE2-blocking antibody on sVNT (expanded)**			
N	119	59	
GM% inhibition (95% CI)	15.0 (15.0-15.0)	15.0 (15.0-15.0)	NA
%positive (≥ LOQ at 30%)	0.0	0.0	> 0.9999

SARS-CoV-2 severe acute respiratory syndrome coronavirus 2, *S* spike protein, *RBD* receptor-binding domain, *IgG* immunoglobulin G, *ELISA* enzyme-linked immunosorbent assay, *N* nucleocapsid protein, *GM* geometric mean, *OD* optical density, *LOD* limit of detection, *LOQ* limit of quantification, *CI* confidence interval, *ACE2* angiotensin-converting enzyme 2, *sVNT* surrogate virus neutralization test, *NA* not applicable. *P* values compare the proportion of positive responses between intramuscular and intradermal administration by Fisher's exact test

Supplementary Table 3. Humoral immunogenicity outcomes against wild type SARS-CoV-2 post-dose 2 and post-dose 3 of CoronaVac in the expanded analysis population

Variables	Intramuscular		Intradermal	
	2 doses	3 doses	2 doses	3 doses
**S IgG on ELISA**				
N	116	77	47	37
GM OD450 value (95% CI)	0.536 (0.493-0.582)	0.872 (0.785-0.968)	0.634 (0.562-0.716)	1.050 (0.983-1.120)
%positive (≥ LOD at 0.3)	94.0	97.4	95.7, *P* > 0.9999	100, *P* > 0.9999
**S-RBD IgG on ELISA**				
N	119	82	59	43
GM OD450 value (95% CI)	1.200 (1.100-1.310)	1.69 (1.600-1.780)	2.15 (2.030-2.280)	2.43 (2.310-2.560)
%positive (≥ LOD at 0.5)	96.6	100.0	100.0, *P* = 0.303	100.0, *P* > 0.9999
**S-RBD ACE2-blocking antibody on sVNT**				
N	119	82	59	43
GM% inhibition (95% CI)	71.2 (66.7-76.0)	84.0 (81.0-87.1)	78.2 (74.5-82.1)	90.7 (87.5-94.0)
%positive (≥ LOQ at 30%)	96.6	100.0	100.0, *P*=0.303	100.0, *P* > 0.9999
**Neutralizing antibody on PRNT**				
N	119	82	59	43
GM PRNT90 (95% CI)	9.8 (8.7-11.1)	18.2 (14.5-22.9)	10.9 (8.39-14.1)	37.5 (26.8-52.4)
%positive (≥ LOD at 10)	65.6	80.5	62.7, *P* = 0.741	97.7, *P* = 0.0061
GM PRNT50 (95% CI)	26.8 (23.0-31.1)	54.2 (44.3-66.5)	30.5 (24.1-38.6)	105.0 (78.9-140.0)
%positive (≥ LOD at 10)	96.6	100.0	98.3, *P* > 0.9999	100, *P* > 0.9999
**S IgG avidity on ELISA**				
N	109	75	45	37
GM avidity index (95% CI)	20.5 (19.1-22.1)	35.6 (32.5-39.1)	7.0 (5.0-9.6)	52.6 (47.7-58.1)
**S IgG FcγRIIIa-binding on ELISA**				
N	116	77	47	37
GM OD450 value (95% CI)	0.749 (0.649-0.864)	1.300 (1.140-1.490)	1.100 (0.955-1.270)	1.780 (1.700-1.870)
%positive (≥ LOD at 0.28)	87.1	97.4	95.7, *P* = 0.156	100.0, *P* > 0.9999

SARS-CoV-2 severe acute respiratory syndrome coronavirus 2, *S* spike protein, *N* nucleocapsid protein, *IgG* immunoglobulin G, *ELISA* enzyme-linked immunosorbent assay, *IgG* immunoglobulin G, *GM* geometric mean, *OD* optical density, *LOD* limit of detection, *LOQ* limit of quantification, *CI* confidence interval, *ACE2* angiotensin-converting enzyme 2, *sVNT* surrogate virus neutralization test, *PRNT* plaque reduction neutralization titer, *FcγRIIIa* Fc gamma receptor III-a. *P* values compare the proportion of positive responses between intramuscular and intradermal administration by Fisher's exact test

Supplementary Table 4. Cellular immunogenicity outcomes against wild type SARS-CoV-2 S, N and M peptide pools at baseline in the evaluable and expanded analysis populations

Variables	Intramuscular	Intradermal	*P*
	Pre-dose 1	Pre-dose 1	
**Total SNM-specific T-cell responses on flow cytometry**			
N	59	47	
GM% IFN-γ^+CD4$^+$ T cells (95% CI)	0.016 (0.012-0.021)	0.022 (0.014-0.032)	0.177
%positive (≥ cut-off at 0.0075%)	83.3	81.3	0.804
GM% IL-2$^+$CD4$^+$ T cells (95% CI)	0.015 (0.012-0.018)	0.019 (0.013-0.027)	0.211
%positive (≥ cut-off at 0.0075%)	83.3	79.2	0.624
GM% IFN-γ^+CD8$^+$ T cells (95% CI)	0.016 (0.012-0.022)	0.022 (0.015-0.033)	0.225
%positive (≥ cut-off at 0.0075%)	65.0	62.5	0.842
GM% IL-2$^+$CD8$^+$ T cells (95% CI)	0.014 (0.012-0.017)	0.023 (0.015-0.034)	0.014
%positive (≥ cut-off at 0.0075%)	58.3	68.8	0.318
**S-specific T-cell responses on flow cytometry**			
N	60	48	
GM% IFN-γ^+CD4$^+$ T cells (95% CI)	0.006 (0.004-0.008)	0.005 (0.003-0.007)	0.559
%positive (≥ cut-off at 0.0075%)	35.0	25.0	2.298
GM% IL-2$^+$CD4$^+$ T cells (95% CI)	0.005 (0.004-0.007)	0.004 (0.003-0.006)	0.131
%positive (≥ cut-off at 0.0075%)	48.3	16.7	0.001
GM% IFN-γ^+CD8$^+$ T cells (95% CI)	0.004 (0.003-0.005)	0.005 (0.003-0.007)	0.449
%positive (≥ cut-off at 0.0075%)	16.7	22.9	0,469
GM% IL-2$^+$CD8$^+$ T cells (95% CI)	0.004 (0.003-0.005)	0.004 (0.003-0.006)	0.916
%positive (≥ cut-off at 0.0075%)	26.7	20.8	0.507
**N-specific T-cell responses on flow cytometry**			
N	59	47	
GM% IFN-γ^+CD4$^+$ T cells (95% CI)	0.003 (0.002-0.004)	0.005 (0.004-0.008)	0.006
%positive (≥ cut-off at 0.0075%)	10.2	27.7	0.024
GM% IL-2$^+$CD4$^+$ T cells (95% CI)	0.003 (0.003-0.004)	0.005 (0.003-0.007)	0.053
%positive (≥ cut-off at 0.0075%)	18.6	23.4	0.632
GM% IFN-γ^+CD8$^+$ T cells (95% CI)	0.004 (0.003-0.005)	0.006 (0.004-0.010)	0.096
%positive (≥ cut-off at 0.0075%)	17.0	27.7	0.237
GM% IL-2$^+$CD8$^+$ T cells (95% CI)	0.003 (0.003-0.004)	0.007 (0.003-0.005)	0.001
%positive (≥ cut-off at 0.0075%)	17.0	36.2	0.027
**M-specific T-cell responses on flow cytometry**			
N	59	47	
GM% IFN-γ^+CD4$^+$ T cells (95% CI)	0.004 (0.003-0.006)	0.005 (0.003-0.006)	0.679
%positive (≥ cut-off at 0.0075%)	23.3	23.4	> 0.9999
GM% IL-2$^+$CD4$^+$ T cells (95% CI)	0.004 (0.003-0.005)	0.004 (0.003-0.005)	0.947
%positive (≥ cut-off at 0.0075%)	32.3	19.2	0.183
GM% IFN-γ^+CD8$^+$ T cells (95% CI)	0.004 (0.003-0.005)	0.004 (0.003-0.006)	0.703
%positive (≥ cut-off at 0.0075%)	17,9	14.9	> 0.9999
GM% IL-2$^+$CD8$^+$ T cells (95% CI)	0.004 (0.003-0.005)	0.004 (0.003-0.006)	0.819
%positive (≥ cut-off at 0.0075%)	33.9	19.2	0,125

SARS-CoV-2 severe acute respiratory syndrome coronavirus 2, *S* spike, *N* nucleocapsid protein, *M* membrane protein, *GM* geometric mean, *IFN-γ* interferon-gamma, *IL-2* interleukin-2, *CI* confidence interval. *P* values compare the proportion of positive responses between intramuscular and intradermal administration by Fisher's exact test

Supplementary Table 5. Cellular immunogenicity outcomes against wild type SARS-CoV-2 S, N and M peptide pools post-dose 2 and post-dose 3 of CoronaVac in the expanded analysis population

Variables	Intramuscular		Intradermal	
	2 doses	3 doses	2 doses	3 doses
**Total S, N, M-specific T-cell responses on flow cytometry**				
N	60	69	48	41
GM% IFN-γ^+CD4$^+$ T cells (95% CI)	0.058 (0.040-0.083)	0.068 (0.044-0.104)	0.107 (0.063-0.183)	0.107 (0.056-0.204)
%positive (≥ cut-off at 0.0075%)	83.3	73.9	81.3, *P* = 0.804	78.1, *P* = 0.656
GM% IL-2$^+$CD4$^+$ T cells (95% CI)	0.040 (0.030-0.052)	0.079 (0.055-0.112)	0.112 (0.068-0.183)	0.142 (0.078-0.258)
%positive (≥ cut-off at 0.0075%)	83.3	81.2	79.2, *P* = 0.624	80.5, *P* > 0.9999
GM% IFN-γ^+CD8$^+$ T cells (95% CI)	0.050 (0.033-0.077)	0.064 (0.038-0.107)	0.059 (0.033-0.104)	0.064 (0.033-0.124)
%positive (≥ cut-off at 0.0075%)	65.0	59.4	62.5, *P* = 0.842	63.4, *P* = 0.692
GM% IL-2$^+$CD8$^+$ T cells (95% CI)	0.017 (0.014-0.022)	0.039 (0.026-0.057)	0.050 (0.031-0.803)	0.067 (0.035-0.127)
%positive (≥ cut-off at 0.0075%)	58.3	60.9	68.8, *P* = 0.318	65.9, *P* = 0.685
**S-specific T-cell responses on flow cytometry**				
N	60	70	48	42
GM% IFN-γ^+CD4$^+$ T cells (95% CI)	0.023 (0.015-0.036)	0.015 (0.010-0.024)	0.022 (0.012-0.040)	0.027 (0.014-0.051)
%positive (≥ cut-off at 0.005%)	70.0	54.3	58.3, *P* = 0.229	69.1, *P* = 0.164
GM% IL-2$^+$CD4$^+$ T cells (95% CI)	0.015 (0.011-0.020)	0.018 (0.011-0.027)	0.020 (0.012-0.035)	0.032 (0.017-0.059)
%positive (≥ cut-off at 0.005%)	73.3	60.0	60.4, *P* = 0.214	69.1, *P* = 0.419
GM% IFN-γ^+CD8$^+$ T cells (95% CI)	0.014 (0.009-0.024)	0.017 (0.010-0.029)	0.014 (0.008-0.025)	0.010 (0.006-0.0197)
%positive (≥ cut-off at 0.005%)	48.3	48.6	45.8, *P* = 0.848	40.5, *P* = 0.439
GM% IL-2$^+$CD8$^+$ T cells (95% CI)	0.006 (0.005-0.008)	0.010 (0.006-0.015)	0.012 (0.007-0.020)	0.010 (0.006-0.018)
%positive (≥ cut-off at 0.005%)	48.3	45.7	52.1, *P* = 0.847	41.5, *P* = 0.696
**N-specific T-cell responses on flow cytometry**				
N	60	69	48	41
GM% IFN-γ^+CD4$^+$ T cells (95% CI)	0.011 (0.008-0.017)	0.013 (0.008-0.022)	0.022 (0.011-0.043)	0.022 (0.011-0.044)
%positive (≥ cut-off at 0.005%)	55.0	50.7	54.2, *P* > 0.9999	58.5, *P* = 0.438
GM% IL-2$^+$CD4$^+$ T cells (95% CI)	0.001 (0.009-0.018)	0.019 (0.012-0.029)	0.028 (0.015-0.053)	0.028 (0.014-0.055)
%positive (≥ cut-off at 0.005%)	66.7	60.9	60.4, *P* = 0.549	63.4, *P* = 0.841
GM% IFN-γ^+CD8$^+$ T cells (95% CI)	0.008 (0.005-0.012)	0.013 (0.008-0.023)	0.016 (0.008-0.031)	0.012 (0.006-0.025)
%positive (≥ cut-off at 0.005%)	31.7	39.1	43.8, *P* = 0.232	39.0, *P* > 0.9999
GM% IL-2$^+$CD8$^+$ T cells (95% CI)	0.004 (0.003-0.005)	0.009 (0.006-0.014)	0.014 (0.008-0.024)	0.014 (0.007-0.026)
%positive (≥ cut-off at 0.005%)	28.3	37.7	47.9, *P* = 0.046	48.8, *P* = 0.318
**M-specific T-cell responses on flow cytometry**				
N	60	70	48	41
GM% IFN-γ^+CD4$^+$ T cells (95% CI)	0.007 (0.005-0.010)	0.006 (0.004-0.010)	0.008 (0.005-0.014)	0.011 (0.005-0.022)
%positive (≥ cut-off at 0.005%)	36.7	25.7	35.4, *P* > 0.9999	34.2, *P* = 0.389
GM% IL-2$^+$CD4$^+$ T cells (95% CI)	0.006 (0.004-0.007)	0.006 (0.004-0.009)	0.011 (0.006-0.019)	0.014 (0.007-0.029)
%positive (≥ cut-off at 0.005%)	46.7	27.1	41.7, *P* = 0.698	46.3, *P* = 0.061
GM% IFN-γ^+CD8$^+$ T cells (95% CI)	0.006 (0.004-0.009)	0.004 (0.003-0.007)	0.007 (0.004-0.011)	0.012 (0.006-0.023)
%positive (≥ cut-off at 0.005%)	25.0	11.4	29.2, *P* = 0.667	41.5, *P* = 0.0004
GM% IL-2$^+$CD8$^+$ T cells (95% CI)	0.004 (0.003-0.005)	0.004 (0.003-0.006)	0.005 (0.004-0.008)	0.012 (0.006-0.023)
%positive (≥ cut-off at 0.005%)	23.3	15.7	27.1, *P* = 0.662	46.3, *P* = 0.0008

SARS-CoV-2 severe acute respiratory syndrome coronavirus 2, *S* spike, *N* nucleocapsid protein, *M* membrane protein, *GM* geometric mean, *IFN-γ* interferon-gamma, *IL-2* interleukin-2, *CI* confidence interval. *P* values compare the proportion of positive responses between intramuscular and intradermal administration by Fisher's exact test

**Supplementary Table 6.** Unsolicited adverse events within 28 days of vaccination in the healthy safety population			
**Variables**	**Intramuscular (*N* = 123)**	**Intradermal (*N* = 59)**	**Overall (*N* = 182)**
**Summary of adverse events and severe adverse events**			
Any adverse event	8 (0.065)	5 (0.085)	13 (0.071)
Grade 1	8 (0.065)	4 (0.068)	12 (0.066)
Grade 2	0 (0.000)	0 (0.000)	0 (0.000)
Grade 3	0 (0.000)	1 (0.017)	1 (0.005)
Severe	0 (0.000)	0 (0.000)	0 (0.000)
Data are number of events (events per participant). *N* total number of participants in the healthy safety population			
